# Electron transfer in an acidophilic bacterium: interaction between a diheme cytochrome and a cupredoxin†
†Electronic supplementary information (ESI) available: N-Terminal sequencing, distribution of charges and dipole moments of the proteins, and stability and modelling of the voltammetric signals. See DOI: 10.1039/c8sc01615a


**DOI:** 10.1039/c8sc01615a

**Published:** 2018-05-01

**Authors:** X. Wang, M. Roger, R. Clément, S. Lecomte, F. Biaso, L. A. Abriata, P. Mansuelle, I. Mazurenko, M. T. Giudici-Orticoni, E. Lojou, M. Ilbert

**Affiliations:** a Aix Marseille Univ , CNRS , IMM , BIP , UMR 7281 , 31 Chemin Aiguier , 13009 Marseille , France . Email: lojou@imm.cnrs.fr ; Email: milbert@imm.cnrs.fr; b School of Life Sciences , University of Dundee , Dundee , DD1 5EH , Scotland , UK; c Institute for Chemistry and Biology of Membrane and Nano-objects , Allée Geoffroy St Hilaire , 33600 Pessac , France; d Laboratory for Biomolecular Modeling , École Polytechnique Fédérale de Lausanne and Swiss Institute of Bioinformatics , AAB014, Station 19 , 1015 Lausanne , Switzerland; e Aix Marseille Univ , CNRS , Institut de Microbiologie de la Méditerranée , FR 3479, Plate-forme Protéomique, Marseille Protéomique (MaP), B.P. 71 , 13402 Marseille Cedex 20 , France; f School of Biomedical Sciences , Leeds , LS2 9JT , UK

## Abstract

*Acidithiobacillus ferrooxidans*, a chemolithoautotrophic Gram-negative bacterium, has a remarkable ability to obtain energy from ferrous iron oxidation at pH 2. Several metalloproteins have been described as being involved in this respiratory chain coupling iron oxidation with oxygen reduction. However, their properties and physiological functions remain largely unknown, preventing a clear understanding of the global mechanism. In this work, we focus on two metalloproteins of this respiratory pathway, a diheme cytochrome c_4_ (Cyt c_4_) and a green copper protein (AcoP) of unknown function. We first demonstrate the formation of a complex between these two purified proteins, which allows homogeneous intermolecular electron-transfer in solution. We then mimic the physiological interaction between the two partners by replacing one at a time with electrodes displaying different chemical functionalities. From the electrochemical behavior of individual proteins, we show that, while electron transfer on AcoP requires weak electrostatic interaction, electron transfer on Cyt c_4_ tolerates different charge and hydrophobicity conditions, suggesting a pivotal role of this protein in the metabolic chain. The electrochemical study of the proteins incubated together demonstrates an intermolecular electron transfer involving the protein complex, in which AcoP is reduced through the high potential heme of Cyt c_4_. Modelling of the electrochemical signals at different scan rates allows us to estimate the rate constant of this intermolecular electron transfer in the range of a few s^–1^. Possible routes for electron transfer in the acidophilic bacterium are deduced.

## Abstract 2

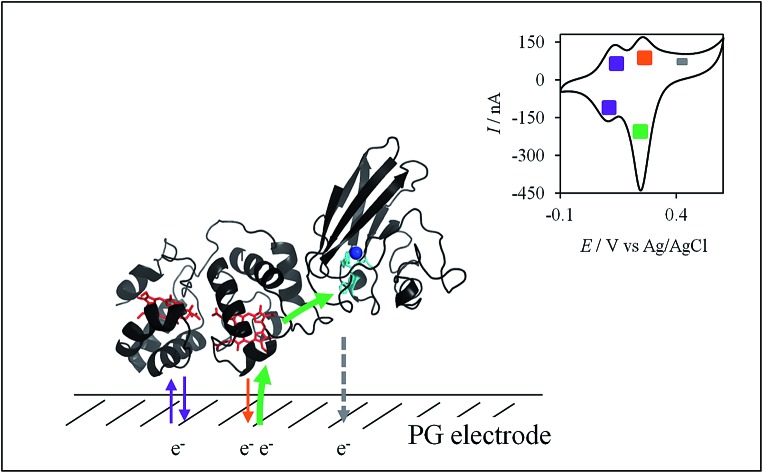
Electrochemical studies of diheme cytochrome/cupredoxin complexes provide new insights into the electron transfer pathway in an acidophilic bacterium.

## Introduction

Biodiversity is an extraordinary source of microorganisms displaying unusual features such as resistance to high temperatures, high pressures, high salinity, extreme pHs, *etc.* Although these organisms were identified many years ago, the molecular factors that allow them to survive and grow under such extreme conditions are far from being completely understood. Extreme acidophiles grow optimally at an external pH of 3 or less. Their ability to survive such drastic environments has aroused a great deal of interest, from both a fundamental and an application point of view.^1^*Acidithiobacillus ferrooxidans* is one of the best studied model organisms and has been used for a long time in bioleaching mine processes and more recently in microbial fuel cells, thanks to its ability to gain energy through the oxidation of ferrous iron at pH as low as 2, thus allowing overcoming the issue of proton availability required to construct an efficient biocathode.^2^,^3^ It has been clearly shown that Fe^2+^ oxidation takes place outside the bacterial cell, while the electrons generated are driven into the periplasmic compartment *via* electron shuttling to the inner membrane where O_2_ reduction takes place.^4^

**Scheme 1:**
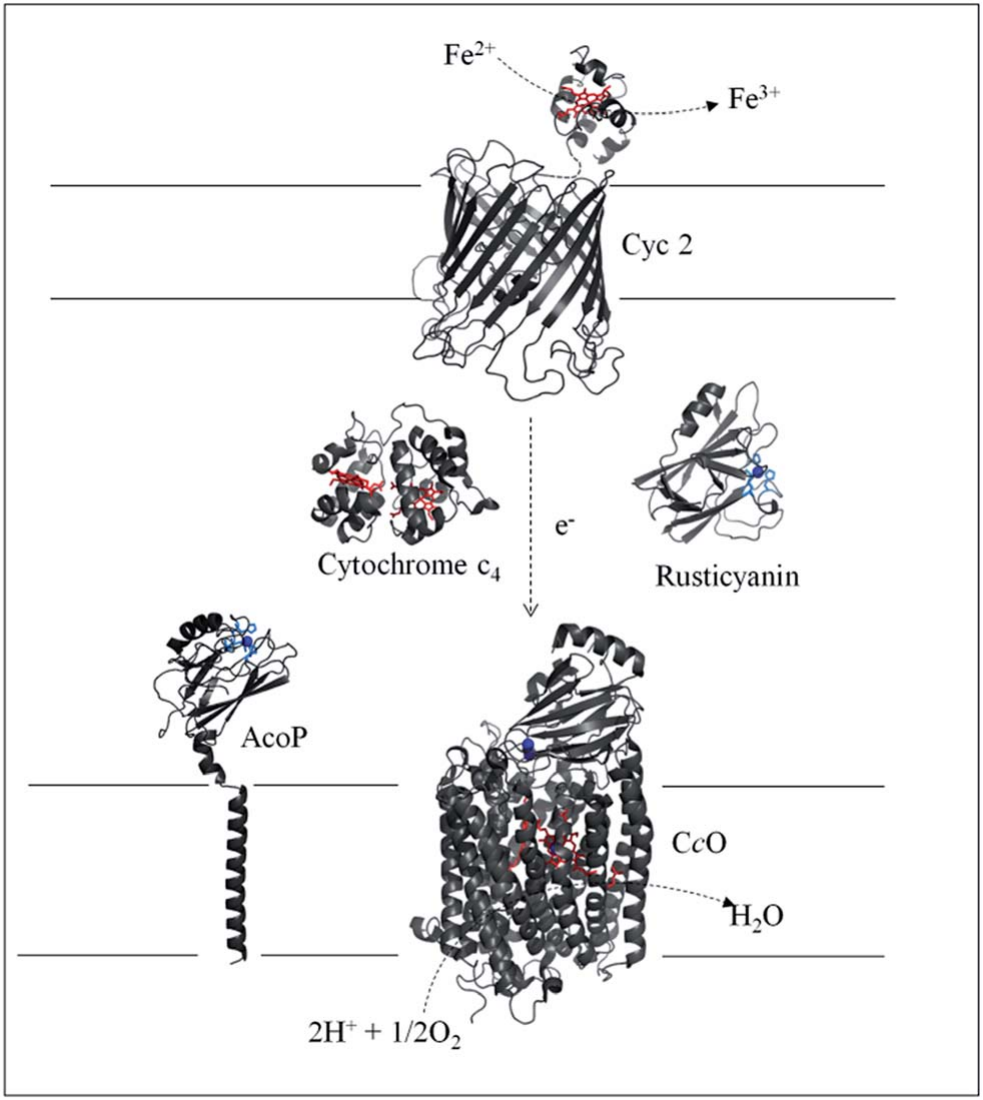
Iron respiratory chain of *Acidithiobacillus ferrooxidans*. Proteins known to be involved in the iron respiratory chain of *A. ferrooxidans* are depicted using Pymol Software. The structures of Cyt c_4_ (PDB: ; 1h1o) and Rus (PDB: ; 1RCY) are known. C*c*O of *Paracoccus denitrificans* is depicted (PDB: ; 3HB3) as an enzyme closely related to C*c*O of *A. ferrooxidans*. The structure of AcoP has been predicted using the Phyre 2 server and the transmembrane segment has been added. The structure of Cyc 2 was predicted.^50^

Several metalloproteins involved in this metabolic chain have already been isolated (Scheme 1), and details on the components have been reported in comprehensive reviews.^5^,^6^ The primary electron acceptor, cytochrome Cyc2, is an outer-membrane monoheme *c*-type cytochrome. Then periplasmic proteins, including a blue copper protein, rusticyanin (Rus), and a diheme *c*-type cytochrome (Cyt c_4_), have been shown to transfer electrons to the terminal electron acceptor, cytochrome *c* oxidase (C*c*O), an inner-membrane protein belonging to the subgroup of heme-copper O_2_ reductases.^7^ All these proteins have in common a high value of their redox potentials,^5^ which fits with the redox potential of the Fe^3+^/Fe^2+^ redox couple at acidic pHs.^8^ A docking model suggests that Cyt c_4_ acts as a wire between Rus and C*c*O, with the heme of the highest potential (Heme_H_) in interaction with Rus, and the heme of the lowest potential (Heme_L_) in contact with C*c*O.^9^ Nevertheless, different models have been proposed to describe the full electron transfer (ET) pathway, and divergence exists concerning the sequential ET pathway between the proteins in the chain.^10^–^12^

Another copper binding protein, AcoP short for “Acidophile Cytochrome *c* Oxidase Partner”, has been found to be directly involved in the respiratory chain.^13^ It has been shown that AcoP copurifies with C*c*O, an enzyme which, in other organisms, is well known to receive its electron from a soluble cytochrome *c.* Most studies have consequently assumed a direct ET between Cyt c_4_ and C*c*O in *A. ferrooxidans*, not taking into account AcoP.^6^,^12^ The presence of AcoP as an additional copper metalloprotein in tight interaction with C*c*O raises the question of its role in the respiratory chain. Its genetic organization confirms its involvement in the respiratory chain as the *acoP* gene is in an operon with the genes encoding the entire pathway.^5^ The presence of a copper site in AcoP may suggest a function beyond the stabilization effect on C*c*O activity in an acidic environment.^13^ Preliminary observations proposed a potential interaction between purified AcoP and Cyt c_4_.^13^ In addition, AcoP's intrinsic properties have recently been fully characterized and point out that it is a novel member of the cupredoxin family with a green copper centre, also called a type 1.5 copper centre, displaying the highest redox potential described to date for this subclass (+350 mV at pH 4.8 *vs.* Ag/AgCl).^14^,^15^ On the basis of its high redox potential and its cupredoxin fold, well suited for optimized ET, a role of AcoP as an electron shuttle might be envisioned, although it has not been demonstrated so far.

Electrochemical techniques are the methods of choice to study the thermodynamics and kinetics of ET processes involved in energy chains.^16^,^17^ ET between various types of electrodes, including carbon or gold surfaces, and small redox proteins such as heme,^18^–^20^ copper^21^ or iron–sulfur clusters^22^ containing proteins, was investigated early on to determine the key factors allowing the electron exchange at the interface. Intramolecular ET between several redox sites in the same protein has also been reported. In the cases where two redox sites are present, thanks to a specific orientation of one site over the other on the electrode, specific electrochemical signals have helped in the quantification of both the interfacial and the intramolecular ET.^23^,^24^ These preliminary studies have served the further quantification of the intermolecular ET within the transitory complex formed between an enzyme and its physiological partner. As examples among many others, second order rate constants between polyheme cytochrome c_3_ and hydrogenases,^25^,^26^ or nitrite reductases^27^ and azurin^28^ or cytochromes^29^ have been obtained by recording the current response to a potential step at electrochemical interfaces. Beyond this, all these studies opened the way for the study of direct catalysis using enzymes at electrochemical interfaces.^30^,^31^

In this paper, we focus on the interaction between AcoP and one of its putative partners in the ET chain, Cyt c_4_, two proteins operating in the pH range of 2.5–3 in the periplasmic compartment.^5^ To our knowledge, very few electrochemical studies have been reported on the interaction between two proteins interacting in an ET pathway even though not directly involved in an enzymatic process. One relevant but rare example is the early voltammetric study of the intermolecular ET between cytochrome *c* and cytochrome *b*_5_, two proteins shown to interact through a protein–protein ET complex.^32^ Thanks to the purification to the homogeneity of both AcoP and Cyt *c*_4_ from *A. ferrooxidans*, we now have the opportunity to investigate an additional ET chain by electrochemistry. In the work presented here, we first used biochemical techniques to demonstrate the occurrence of a complex between AcoP and Cyt c_4_ which allows ET in a homogeneous aqueous phase. To reveal the molecular determinants of a protein–protein interaction favourable to ET in the acidophilic chain of *A. ferrooxidans*, we then analysed the interfacial ET between each individual protein and an electrode, with the aim of mimicking partner–partner interaction. For this purpose, the surface chemistry of the electrode was modified and further modulated by pH and ionic strength. We finally took advantage of the knowledge of the electrode surface chemistry required for the interfacial ET on an individual protein, with the ultimate goal of analysing the consequence of the formation of a complex between AcoP and Cyt c_4_ on the electrochemical signal. We discuss the occurrence of intermolecular ET between the two proteins at the electrochemical interface. This ET process suggests new insights into the ET pathway allowing *A. ferrooxidans* to grow under acidic conditions.

## Results and discussion

### Complex formation and intermolecular electron transfer between Cyt c_4_ and AcoP in solution

**Fig. 1:**
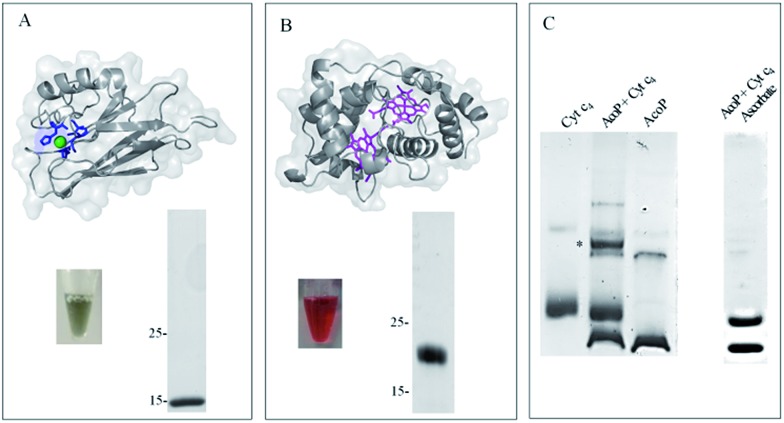
AcoP : Cyt c_4_ complex formation in solution from purified proteins. (A) Structure prediction and purified AcoP in solution or loaded on SDS-PAGE gel. The structure prediction of AcoP using the Phyre 2 server is in agreement with a cupredoxin fold mainly composed of β sheets previously observed by circular dichroism.^14^ The 4 residues identified as copper ligands^15^ are depicted in blue and are found in close proximity to the copper center (green sphere) which validate the model; (B) structure and purified Cyt c_4_ in solution or loaded on SDS-PAGE gel and blue stained. The structure (PDB: ; 1H1O) is shown in grey with the two hemes in magenta; (C) 5 μl of 100 μM Cyt c_4_, AcoP or AcoP : Cyt c_4_ 1 : 1 complex were loaded on a “Modified-native” gel. An additional band labeled (*) is observed when the complex AcoP : Cyt c_4_ migrates on the gel. When mentioned the AcoP : Cyt c_4_ complex was incubated with 700 μM ascorbate before migration.

To demonstrate the formation of a complex between AcoP and Cyt c_4_, we first purified both proteins to homogeneity as shown in Fig. 1.

Both purified proteins were incubated for 12 hours, either separately or together, and subsequently loaded on a “modified-native” gel containing a small amount of SDS (0.0175% *versus* 0.1% in SDS-PAGE gel). As shown in Fig. 1C, in this experimental set-up, a clear additional band, of a higher molecular weight than the individual proteins, and labeled (*) could be visualized when AcoP and Cyt c_4_ were incubated together. This additional band was not seen using SDS-PAGE gel (ESI Fig. S1†). To confirm the presence of both proteins in the band labeled (*), N-terminal sequencing was performed. The results presented in ESI Fig. S2† unequivocally confirm the presence of AcoP and Cyt c_4_ and thus the existence of an observable AcoP/Cyt c_4_ complex. No complex formation occurred between AcoP and Cyt c_4_ in the presence of ascorbate, suggesting that it is dependent on the redox state of the proteins (Fig. 1C).

**Fig. 2:**
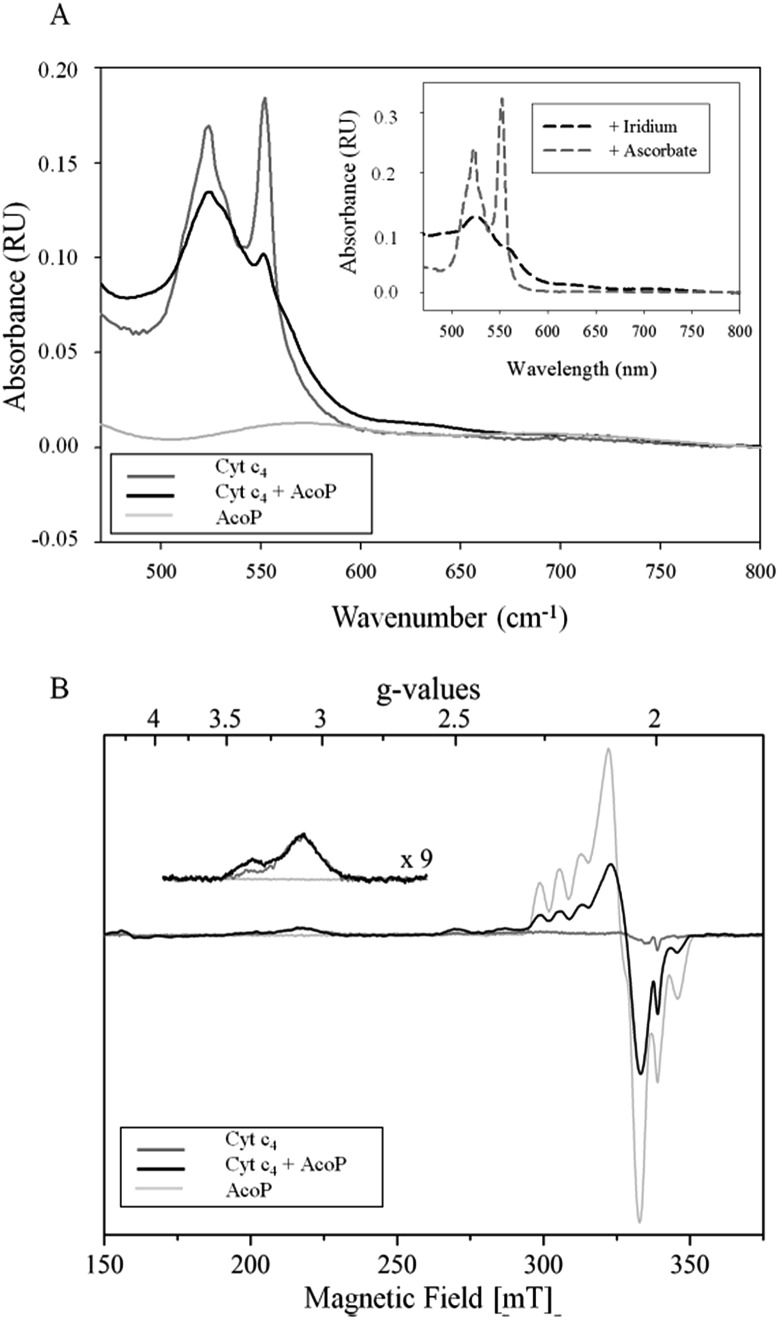
(A) UV-vis spectra of 15 μM AcoP “as prep” (light grey), 15 μM reduced Cyt c_4_ (dark grey), and AcoP/Cyt c_4_ 1 : 1 mix (15 μM of each in the final mixture) (black) in 20 mM NaAC buffer, pH 4.8. Inset: typical spectra of oxidized Cyt c_4_ obtained after the addition of K_2_IrCl_6_ (black dashed line) or of reduced Cyt c_4_ obtained after the addition of ascorbate (grey dashed line); (B) EPR spectra of 100 μM AcoP “as prep” (light grey), 100 μM Cyt c_4_ untreated (dark grey) and AcoP/Cyt c_4_ 1 : 1 mix (100 μM of each in the final mixture) (black) in 20 mM NaAc buffer, pH 4.8. Acquisition parameters: temperature = 15 K, microwave frequency = 9479 MHz, microwave power = 0.2 mW, and modulation amplitude = 2 mT. Inset: magnification of the *g*_z_-peak region of low-spin hemes (microwave power = 4 mW).

The involvement of AcoP in an electron pathway through Cyt c_4_ in solution was then evaluated. Purified Cyt c_4_ in its reduced form (Fig. 2A) was incubated with “as prep” AcoP in an equimolar amount. UV-vis spectroscopy clearly showed Cyt c_4_ oxidation in the presence of AcoP, revealed by the decrease of the β and γ bands of heme *c* groups at 523 and 552 nm, and a net change of the ratio between these two bands (Fig. 2A). For comparison, the spectrum of Cyt c_4_ reduced by ascorbate and oxidized by K_2_IrCl_6_ is shown in the Fig. 2A inset.

Given the low absorption coefficient of AcoP at 570 nm (∼2500 M^–1^ cm^–1^ (ref. 15)) compared to that of Cyt c_4_ at a similar wavelength (*ε*_552 nm_ = 46 000 M^–1^ cm^–1^ (ref. 33)), the concomitant reduction of AcoP cannot be observed using this approach. To observe AcoP and Cyt c_4_ signatures independently, EPR measurements of frozen solutions of Cyt c_4_, AcoP and AcoP/Cyt c_4_ 1 : 1 mix were performed (Fig. 2B). The Cyt c_4_ spectrum exhibits two positive peaks, at *g* = 3.39 and 3.13, corresponding to the *g*_z_-values of oxidized Heme_H_ and Heme_L_, respectively.^34^ The broad signal around *g* = 2.3 can be attributed to the *g*_y_ parts of heme signals. As expected for low-spin ferric heme signals with a maximum *g* value higher than 3, the *g*_x_ contributions are too broad to be detected.^35^ The AcoP spectrum was characteristic of a rhombic Cu(ii) centre with *g*-values equal to 2.193, 2.057 and 2.019, and copper hyperfine coupling constants of 66 × 10^–4^, 12 × 10^–4^ and 65 × 10^–4^ cm^–1^ respectively.^14^ The addition of one equivalent of Cyt c_4_ led to a significant decrease of the AcoP signal, suggesting the reduction of the Cu(ii) site. In addition, the Heme_H_ peak at *g* = 3.39 increases by a factor of 2.4 in intensity, which indicates an oxidation of the Heme_H_ concomitant with the reduction of the AcoP copper site. Thus UV-vis and EPR spectroscopies both demonstrate that ET between AcoP and Cyt c_4_ occurs in solution *in vitro*, most probably through Heme_H_.

### Interfacial electron transfer properties of AcoP

More insights into the conditions and kinetics of complex formation between AcoP and Cyt c_4_ were obtained by using electrochemical techniques. The additional interest of using electrochemistry was that the electrochemical interface could be easily tuned to give access to the key parameters allowing the relationship between interfacial ET and pH, ionic strength, *etc.*, to be investigated. The data obtained by such an investigation could help to define the type of interaction involved in the interfacial ET between the electrode and each protein, and, beyond, between the two proteins if we consider the electrode as one of the partners. We first mimicked the AcoP-partner interaction by studying the electrochemical behavior of AcoP under different experimental conditions at two different electrodes, pyrolytic graphite (PG) and gold, the electrode mimicking its partner, Cyt c_4_.

**Fig. 3:**
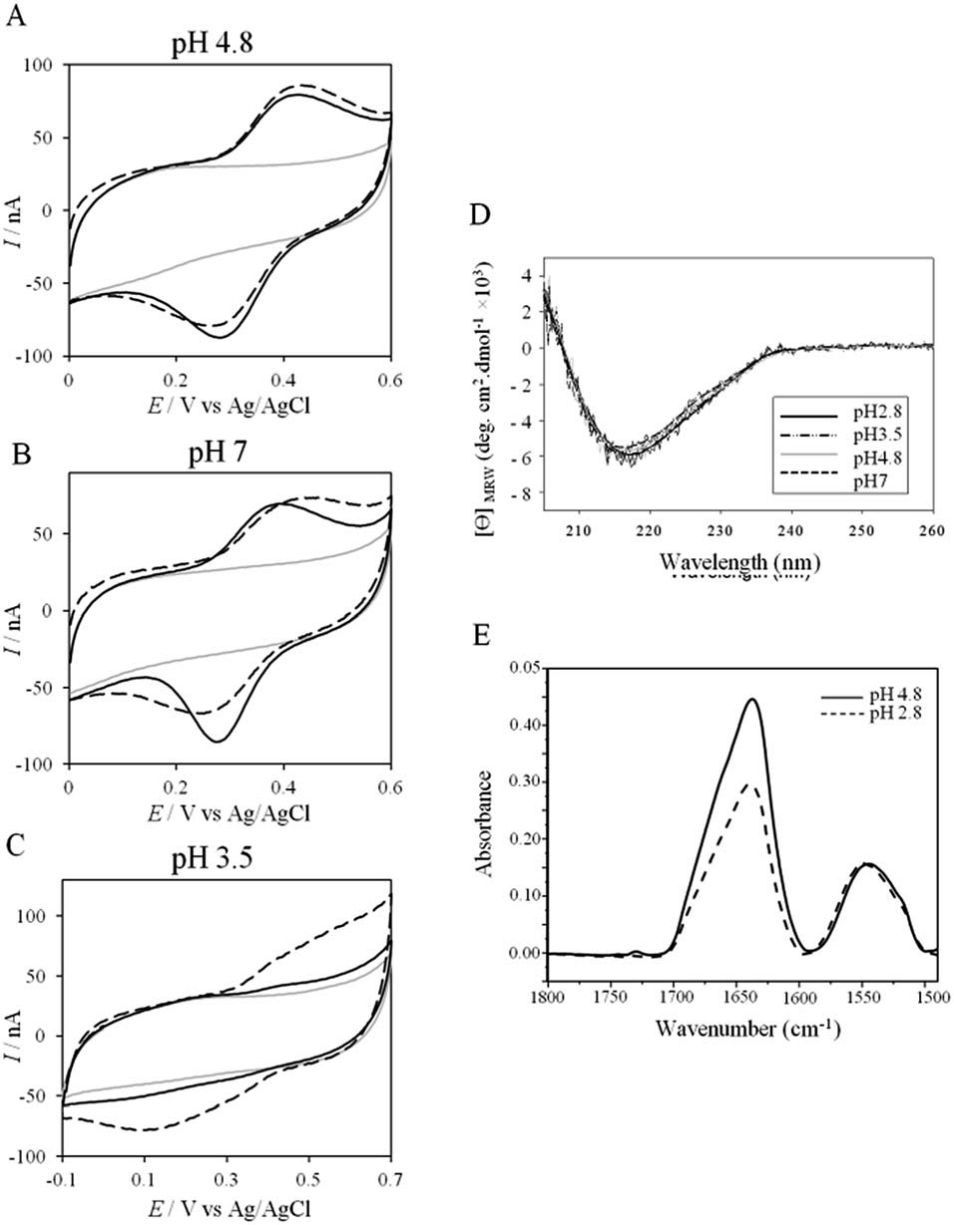
(A–C) CVs of 50 μM AcoP at a PG electrode in the membrane configuration at pH 4.8 (A), pH 7 (B), and pH 3.5 (C) in 20 mM NH_4_AC buffer, before (black solid lines) and after the addition of 200 mM NaCl (black dashed line). *v* = 20 mV s^–1^. Grey solid lines represent the CVs with no protein; (D) CD spectra of AcoP at different pHs in 10 mM NH_4_AC buffer at room temperature; (E) ATR-FTIR spectra of AcoP at 20 μM in 10 mM NH_4_AC at pH 4.8 (solid line) and pH 2.8 (dashed line).

The role of potential electrostatic interactions was first evaluated by studying the electrochemical behavior of AcoP at a PG electrode. Three particular pHs, 3.5, 4.8 and 7, were chosen as they allowed both the global charge of the protein and the charge of the electrode to be tuned. The PG surface carries many carboxyl functions that induce a p*K*_a_ close to 5.^36^ Modifying the electrode at these different pHs enabled positively (pH 3.5) or negatively charged (pH 7) electrodes to be created. AcoP has a theoretical pI of 7.2 and is thus globally positively charged at pH 3.5 and 4.8, and neutral at pH 7. Fig. 3 reports the voltammetric behavior of AcoP entrapped in the thin layer of the membrane electrode configuration at these 3 pHs in 20 mM NH_4_AC buffer before and after the addition of 200 mM NaCl, which was used to screen possible electrostatic interactions.^37^ The relationship between the mean potential and pH is given in ESI Fig. S3.†

At pH 4.8, the one-electron redox wave characteristic of the Cu site in AcoP appears with an average potential of +350 ± 5 mV, in agreement with previous measurements either in solution using UV-vis titration^14^ or at a PG electrode.^15^ The difference between the cathodic and the anodic peaks, Δ*E*_p_, is close to 120 mV at 20 mV s^–1^, denoting a slow ET rate. The addition of up to 200 mM NaCl in the buffer did not induce a change either in the peak current or in Δ*E*_p_. When cyclic voltammetry (CV) was performed at pH 7, a redox wave at +340 ± 5 mV was observed displaying a Δ*E*_p_ of 90 mV, which was not stable over consecutive cycles (ESI Fig. S3†). The addition of NaCl tended to increase Δ*E*_p_ to 160 mV, but also induced the stability of the signal. Decreasing the pH to 3.5 or lower led to a drastic loss of the reversible redox wave, which hardly reappeared after NaCl addition. The transfer of the AcoP-based electrode from pH 3.5 back to pH 4.8 did not lead to the recovery of the redox signal.

**Fig. 4:**
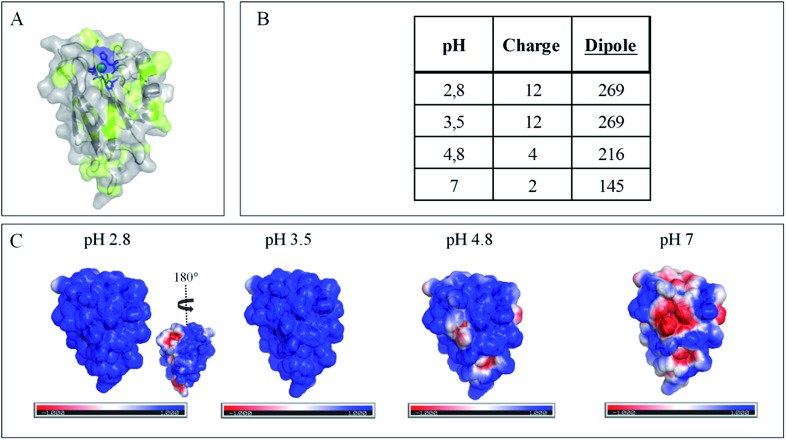
Impact of pH on exposed AcoP amino acids. (A) Hydrophobic surfaces (light green) depicted using Pymol software on the predicted structure of AcoP. (B) Dipole moment of AcoP and the number of charged atoms at different pHs using the Protein Dipole Moments server. (C) Electrostatic charges at the surface of AcoP as a function of pH is shown using Pymol with the following color code: positive charges in blue, negative charges in red, and neutral in white.

The evolution of the CV signal of AcoP as a function of buffer pH and ionic strength can be explained considering the distribution of protein charges at a given pH (Fig. 4), which induces dipole moments of 269, 216 and 145 Debye at pH 3.5, 4.8 and 7, respectively. Since we used a model of the AcoP structure, the exact dipole moments might be slightly different, but we believe that the trend is realistic. These dipole moments are 3–4 times lower than values obtained for proteins known to interact with the electrode surface through electrostatic interactions,^38^,^39^ suggesting that this type of interaction is not the main force driving the interaction of AcoP with the electrode. Accordingly, interfacial ET occurs only under conditions where electrostatic interactions are weak, such as pH 4.8, where AcoP is globally positively charged while PG is neutral, or pH 7, where the protein is globally neutral and the electrode is negatively charged. In the latter case, ET is stabilized by the presence of NaCl, which weakens electrostatic interactions.

The loss of the redox wave at low pHs was unexpected for an acidophilic protein and raises the question of AcoP structural stability on the PG electrode. UV-visible spectra had previously shown that copper geometry was not affected by changes in pH in the range from 3.5 to 7.4.^14^ The present work further demonstrates that even a pH as low as pH 2.8 does not induce any modification of the copper centre (ESI Fig. S4†). Circular Dichroïsm (CD) (Fig. 3D) and ATR FTIR (Fig. 3E) measurements of AcoP solutions at different pHs confirm that the decrease of pH has no effect on the secondary structure of AcoP. CD spectra can be superimposed in the pH range of 2.8–7, and no shift of the amide I band at around 1630 cm^–1^, characteristic of a structure rich in β-sheets, was observed in the ATR FTIR spectra.

**Fig. 5:**
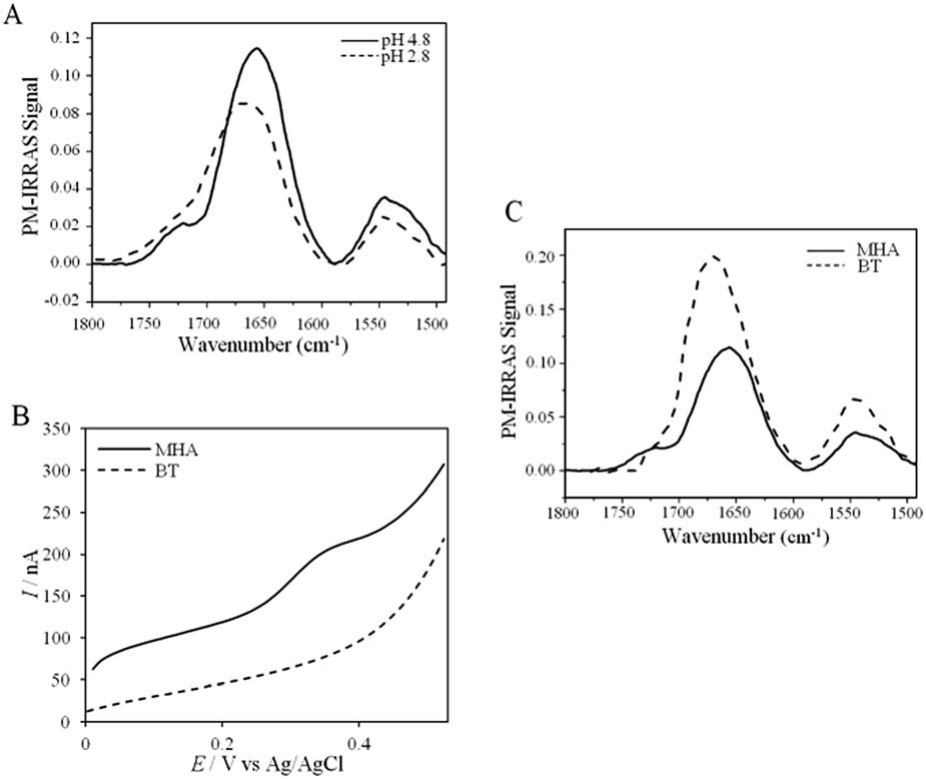
AcoP behavior on SAMs. (A) PMIRRAS spectrum of AcoP at 20 μM adsorbed for 1 h on MHA-Au in 10 mM NH_4_AC at pH 4.8 (solid line) and pH 2.8 (dashed line); (B) SWVs of AcoP adsorbed on MHA (solid line) or BT (dashed line) in 10 mM NH_4_AC at pH 4.8; (C) PMIRRAS spectra of AcoP at 20 μM in 10 mM NH_4_AC at pH 4.8 adsorbed for 1 h on MHA-Au (solid line) or BT-Au (dashed line).

UV-vis, CD and ATR-FTIR measurements underlined that AcoP was able to tolerate a broad range of pH conditions in solution, with no impact on its overall fold. These observations suggest that the absence of interfacial ET at low pH is most probably linked to the interaction with the electrode surface, which irreversibly impacts the native conformation of AcoP. Polarization modulation-infrared reflection-adsorption spectroscopy (PMIRRAS) on a Self-Assembled-Monolayer (SAM) on gold surfaces was used to study any conformational change of the protein upon immobilization on the electrode, especially at low pH. A 6-Mercaptohexanoic acid (MHA)-based SAM (Fig. 5A) was chosen because the induced surface chemistry and p*K*_a_ (p*K*_a_ of MHA as a SAM is close to 6 (ref. 18)) resemble those of PG. The carboxylic groups of the MHA-based SAM are observed at around 1735 cm^–1^. Both amide I and amide II bands appear on PMIRRAS spectra whatever the pH (Fig. 5A), demonstrating that AcoP is readily adsorbed on MHA-based SAMs at the two pHs under investigation. However, the position of the amide I band differs as a function of pH, shifting from 1655 cm^–1^ to 1670 cm^–1^ at pH 4.8 and 2.8, respectively. The typical band at 1655 cm^–1^ corresponds to the amide group involved in α-helices, while the band at 1670 cm^–1^ is assigned to the amide group implicated in turns. As described above, AcoP in solution is mainly structured in β-sheets, with a characteristic wavenumber of 1630 cm^–1^. The first conclusion is that adsorption on MHA-based SAMs induces either a modification of the protein secondary structure or a specific orientation of AcoP with the main contribution of the AcoP α-helix perpendicular to the surface according to PMIRRAS selection rules.^40^ At pH 4.8, because AcoP is still electroactive (Fig. 5B), strong denaturation is unlikely, but instead the maximum observed at 1655 cm^–1^ would reveal an orientation of the AcoP on the surface. At pH 2.8, the AcoP amide I band was shifted from 1655 cm^–1^ to 1670 cm^–1^. At this pH, the loss of electroactivity (Fig. 3) would be favorable for some modification of the secondary structure rather than variation in the orientation of AcoP. In conclusion, AcoP adsorption on hydrophilic surfaces at low pH affects its secondary structure, and hence interfacial ET.

Native AcoP has a transmembrane segment and has been proposed to interact with the membrane bound C*c*O. Having ruled out a major role of electrostatic interactions, we proceeded to test whether hydrophobic interactions could rather drive interfacial ET and consequent complex formation. Fig. 4 underlines some hydrophobic patches on the surface of the protein which could be involved in the partner recognition. In a mimicking way, the putative recognition of AcoP with the electrode surface through hydrophobic interactions was studied on a hydrophobic butane thiol (BT)-based SAM on a gold electrode, on which fast ET on azurin was previously obtained.^28^ No SWV signal was observed with AcoP adsorbed on the BT-SAM (Fig. 5B). PMIRRAS was used to compare the conformation of the protein upon immobilization on the BT-SAM with that on MHA-based SAM surfaces (Fig. 5C). The spectra show that AcoP is also readily adsorbed on a BT-based SAM surface. Changes in the conformation of AcoP can be observed, with a loss of β sheets. The amide *I* position is shifted to 1670 cm^–1^, revealing the presence of a high level of turns in the structure of AcoP adsorbed on the hydrophobic surface. AcoP adsorption on a BT-based SAM electrode might induce a conformational change of the protein, explaining the absence of an electrochemical response on a hydrophobic surface.

The conclusions that can be drawn from electrochemistry and PMIRRAS are that hydrophobic surfaces, or strong electrostatic force between highly positively charged proteins at low pH and hydrophilic surfaces, irreversibly modify the AcoP structure and impair ET to the electrodes. This is in agreement with the fact that periplasmic proteins from acidophiles operate at pH lower than 3 and are highly positively charged (Fig. 4). Under such conditions, electrostatic interactions mostly involve the repulsion between positive charges impairing the ET. This would explain the requirement of weak electrostatic interactions between AcoP and the electrode for efficient ET. It cannot be excluded that hydrophobic interactions might drive the interaction between proteins in such an environment, but would involve small hydrophobic patches. A novel design of electrodes would be required to mimic such surfaces.

### Interfacial ET properties of Cyt c_4_

**Fig. 6:**
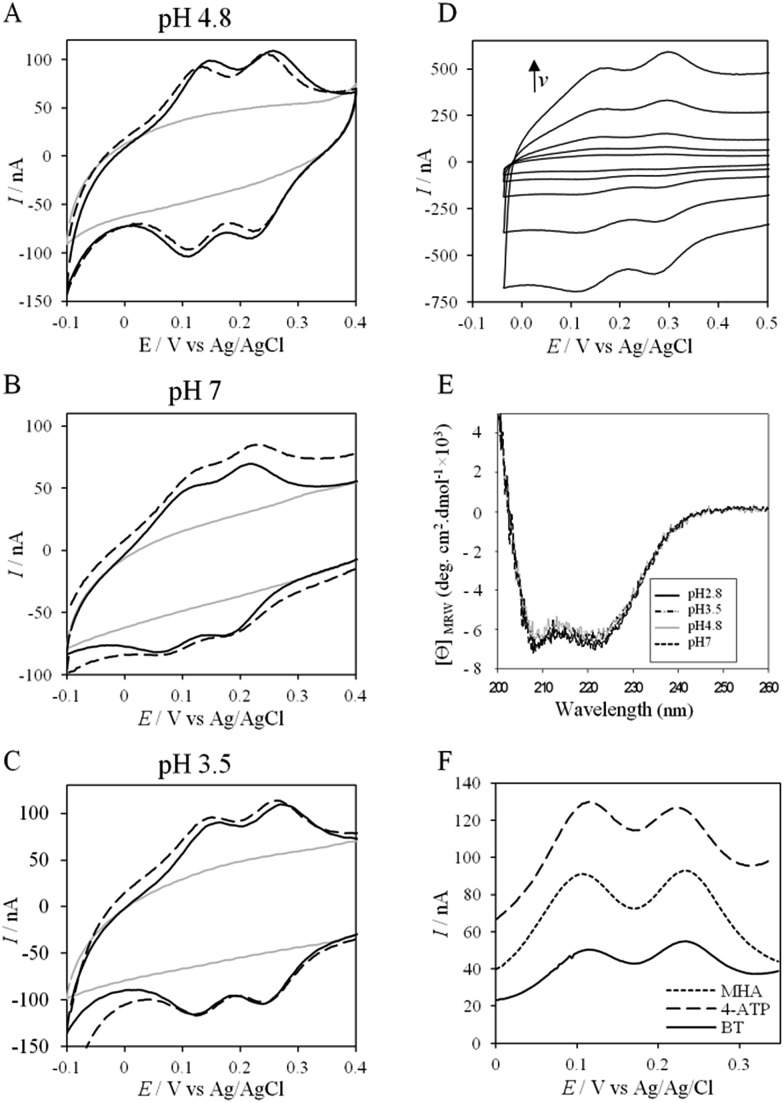
(A–C) CVs of 50 μM Cyt c_4_ at a PG electrode in the membrane configuration at pH 4.8 (A), pH 7 (B), and pH 3.5 (C) in 20 mM NH_4_AC buffer, before (black solid lines) and after the addition of 200 mM NaCl (black dashed line). *v* = 20 mV s^–1^. Grey solid lines represent the CVs with no protein; (D) CV of Cyt c_4_ adsorbed at the PG electrode at 5, 10, 20, 50 and 100 mV s^–1^; (E) CD spectra of Cyt c_4_ at different pHs in NH_4_AC 10 mM buffer at room temperature; (F) SWVs on MHA (short dashed line), 4-ATP (dashed line) and BT (solid line) in 20 mM NH_4_AC buffer pH 4.8.

**Fig. 7:**
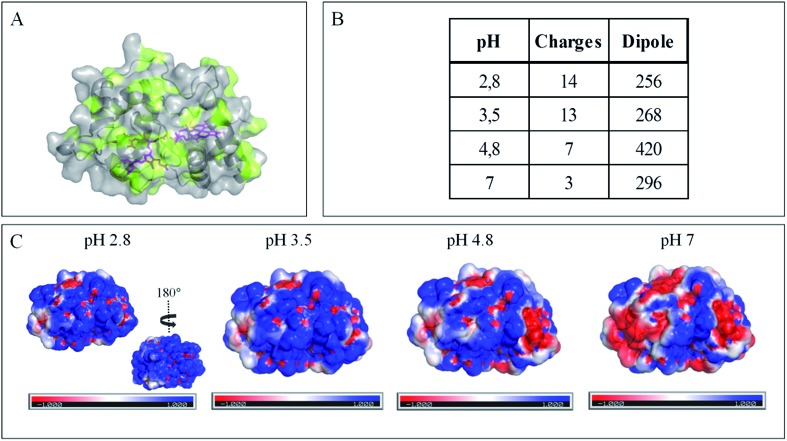
Impact of pH on exposed Cyt c_4_ amino acids. (A) Hydrophobic surfaces (light green) depicted using Pymol software on the predicted structure of Cyt c_4_. (B) Dipole moment of Cyt c_4_ and the number of charged atoms at different pHs obtained using the Protein Dipole Moments Server. (C) Electrostatic charges at the surface of Cyt c_4_ as a function of pH is shown using Pymol with the following color code: positive charges in blue, negative charges in red, and neutral in white.

To further evaluate the factors that can control protein–protein interaction in the metabolic chain of *A. ferrooxidans*, the electrochemical behavior of Cyt c_4_ was studied at the three pHs used previously*, i.e.* pH 3.5, 4.8 and 7, with a PG electrode acting as a partner (Fig. 6). At all these pHs, Cyt c_4_ is globally positively charged (the theoretical pI of Cyt c_4_ is 8) (Fig. 7). At pH 4.8 (Fig. 6A), two redox waves were observed, with mean potentials of +235 ± 5 mV and +125 ± 5 mV, corresponding to the high potential heme, Heme_H_, and the low potential heme, Heme_L_, respectively. These values are in good agreement with the previous ones determined at a gold electrode modified with bis(4-pyridyl)disulfide.^9^ The ratio of peak heights of Heme_H_ and Heme_L_ is close to 1, and Δ*E*_p_ for both processes is around 30 mV at 20 mV s^–1^, showing that the ET rate is fast and equivalent for the two hemes. As with AcoP, the addition of NaCl did not change the profile of the CV. When the membrane was removed from the electrode, the redox waves for Cyt c_4_ persisted (Fig. 6D), showing the stable adsorption of some molecules. From the charge under the redox waves at different scan rates, a surface coverage of 20 ± 2 pmol cm^–2^ was calculated. At more basic and more acid pHs (Fig. 6B and C), the curves for the two redox processes for Heme_H_ and Heme_L_ were still well shaped. Notably at pH 7, the redox signal for Cyt c_4_ was not stable over time, even after the addition of NaCl (ESI Fig. S5†). Using CD and optical spectroscopy we did not see any critical modification of the Cyt c_4_ structure in solution in the pH range of 2.8–7 (Fig. 6E, ESI Fig. S6†). Cyt c_4_ loss of activity on the electrodes at pH 7 might have been due to electrostatic interaction with the negatively charged electrode surface which was far from physiological conditions for acidophilic organisms.

Overall, the occurrence of a redox signature for both hemes, and their equal intensities whatever the pH, and hence whatever the charge of the PG electrode, suggest that Cyt c_4_ cannot be specifically oriented at the electrochemical interface. This is different from what was found for the Cyt c_4_ from *Pseudomonas stutzeri*, which had distinct positive and negative domains surrounding each heme at pH 7 (ESI Fig. S7†), properties that allowed its orientation on a negatively charged SAM electrode with one heme facing the electrode.^23^ The absence of such an orientation of Cyt c_4_ was further assessed by immobilizing the protein on SAM electrodes bearing different charges (Fig. 6F). Well-shaped SWV signals were obtained at negatively charged MHA and positively charged 4-aminothiol (4-ATP) SAM electrodes at pH 4.8, with redox potentials of 230 ± 5 mV and 115 ± 5 mV, corresponding to Heme_H_ and Heme_L_. Cyt c_4_ from *A. ferrooxidans* displays a dipole moment ranging from 256 D to 420 D depending on the pH, half that of *P. stutzeri* Cyt c_4_ at pH 7. A comparison of their surface charges (ESI Fig. S7†) clarifies the non-orientation of *A. ferrooxidans* Cyt c_4_, as positive and negative charges are randomly exposed to the protein surface, in contrast with *P. stutzeri* Cyt c_4_. Interestingly, on a hydrophobic BT-based SAM, the redox waves relative to the two hemes also appeared at their expected redox potentials, demonstrating an efficient ET interaction between Cyt c_4_ and hydrophobic surfaces. This interaction can be easily understood by considering the large hydrophobic areas on the surface of the protein (Fig. 7).

This is also in agreement with molecular dynamic simulations that showed that the interaction between Cyt c_4_ and CoxB, the subunit of CcO transferring the electrons to the catalytic site, was stabilized by hydrophobic residues.^12^ Unlike AcoP, Cyt c_4_ thus appears as a versatile partner that should afford many types of interactions for ET with both hemes. In line with this conclusion, it was recently proposed that Cyt c_4_ from *A. ferrooxidans* would be involved in the anaerobic electron transfer chain coupling Fe^3+^ reduction to sulfur oxidation.^41^ In such a case, Cyt c_4_ would transfer electrons to other partners than C*c*O. Interestingly, versatility has also been demonstrated recently using the diheme cytochrome *c*_550_ from *Thermus thermophilus*.^42^ Whether it is a general property of diheme cytochromes is an interesting open question.

### Electrochemical behavior of a mixture of AcoP and Cyt c_4_

**Fig. 8:**
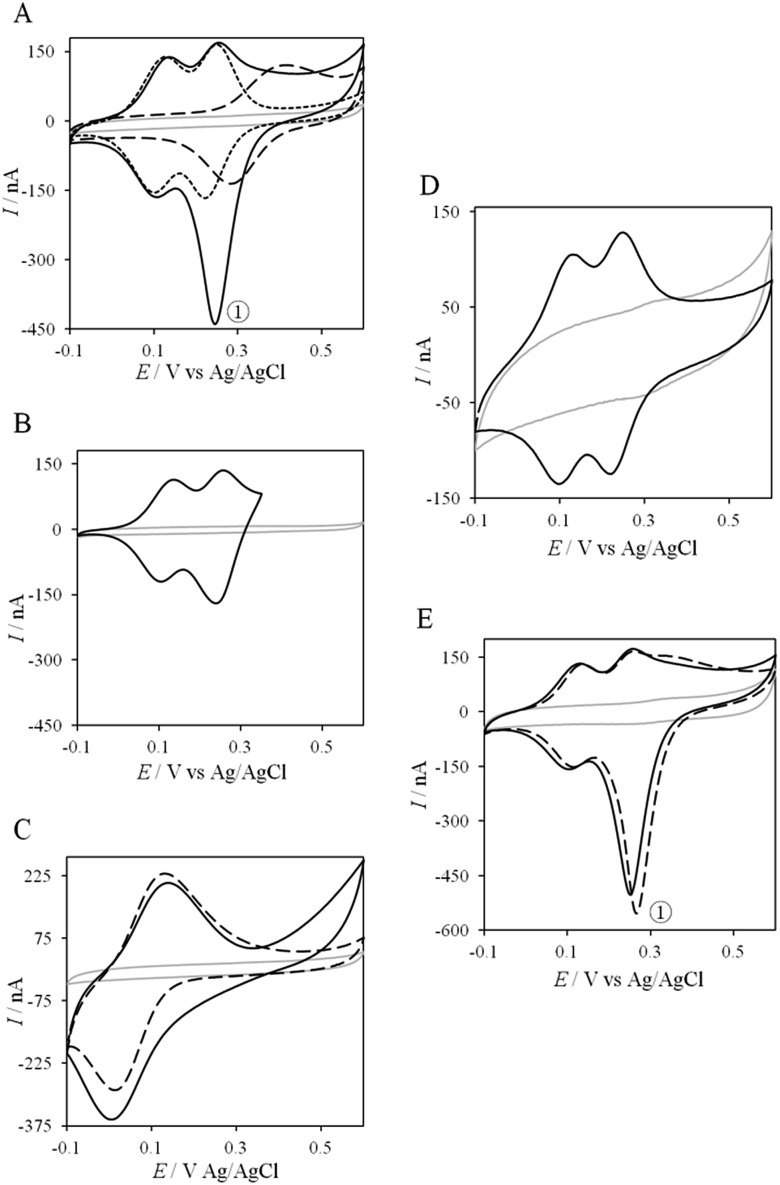
Electrochemical behavior of the AcoP : Cyt c_4_ complex at a PG electrode in the membrane configuration. (A) CV of 100 μM AcoP (dashed line), 100 μM Cyt c_4_ (short dashed line), and the 1 : 1 mixture of AcoP and Cyt c_4_ (100 μM of each in the final mixture) (solid line); (B) CV of the 1 : 1 mixture of AcoP and Cyt c_4_ in a restricted potential window; (C) CV of horse heart Cyt *c* (dashed line) and the 1 : 1 mixture of AcoP and Cyt *c* (solid line); (D) CV of the 1 : 1 mixture of AcoP H166A and Cyt c_4_; (E) CV of the 1 : 1 mixture of AcoP and Cyt c_4_ before (solid line) and after the addition of 400 mM NaCl (dashed line). Peak before (solid line) and after the addition of 400 mM NaCl (dashed line). Peak ① represents the intermolecular ET process. Grey solid lines represent the CVs with no protein. pH 4.8, 20 mM NH represents the intermolecular ET process. Grey solid lines represent the CVs with no protein. pH 4.8, 20 mM NH_4_AC, 20 mV s^–1^.

Thanks to the knowledge of the electrochemical behavior of individual proteins, the kinetics of ET between AcoP and Cyt c_4_ was further studied using electrochemistry. Fig. 8 shows the CVs obtained at 20 mV s^–1^ at the PG electrode in the membrane configuration with a mixture of AcoP and Cyt c_4_ in a 1 : 1 ratio, incubated for 12 h at pH 4.8. The first CV cycle was run in the forward scan from –0.1 V to +0.6 V. The second cycle superimposed onto the first one (not shown). The signals for AcoP and Cyt c_4_ alone under the same experimental conditions are overlaid for comparison. Interesting features can be extracted from these CV experiments. Redox waves corresponding to Heme_L_ in the mixture are similar to those found with Cyt c_4_ alone, both in terms of mean potential and peak height. Such a result demonstrates that the low potential Heme_L_ is not affected by the presence of AcoP. The oxidative wave for Heme_H_ is also conserved in the mixture compared to Cyt c_4_ alone. In contrast, no well-shaped redox signal at the expected potentials for AcoP can be observed in the CV of the mixture, suggesting that heterogeneous interfacial ET for AcoP is slowed down in the presence of Cyt c_4_. However, a reduction wave at a peak potential around +250 mV appears with a marked increase in the peak current compared to either Heme_H_ of Cyt c_4_ or AcoP alone.

The reduction wave at +250 mV, denoted peak The reduction wave at +250 mV, denoted peak ① in the following, strongly decreases when the forward scan of the CV is restricted to +350 mV, in the following, strongly decreases when the forward scan of the CV is restricted to +350 mV, *i.e.*, before the expected oxidation of AcoP (Fig. 8B). Peak ). Peak ① thus involves the reduction of oxidized AcoP. Peak ① does not appear when the same experiment is performed with thus involves the reduction of oxidized AcoP. Peak ). Peak ① thus involves the reduction of oxidized AcoP. Peak ① does not appear when the same experiment is performed with does not appear when the same experiment is performed with *Horse heart* Cyt *c* instead of Cyt c_4_ (Fig. 8C). This last result strongly suggests that peak ). This last result strongly suggests that peak ① is linked to a specific interaction between AcoP and Cyt c is linked to a specific interaction between AcoP and Cyt c_4_. We repeated similar experiments with the mutant AcoP_His166A_ previously shown to have lost its redox properties, even though its secondary structure and copper content were unmodified (Fig. 8D).^13^ Peak Peak ① was absent when AcoP was absent when AcoP_His166A_ was incubated with Cyt c_4_, underlining the importance of a redox active state of AcoP to observe the particular electrochemical behavior of the complex.

**Scheme 2:**
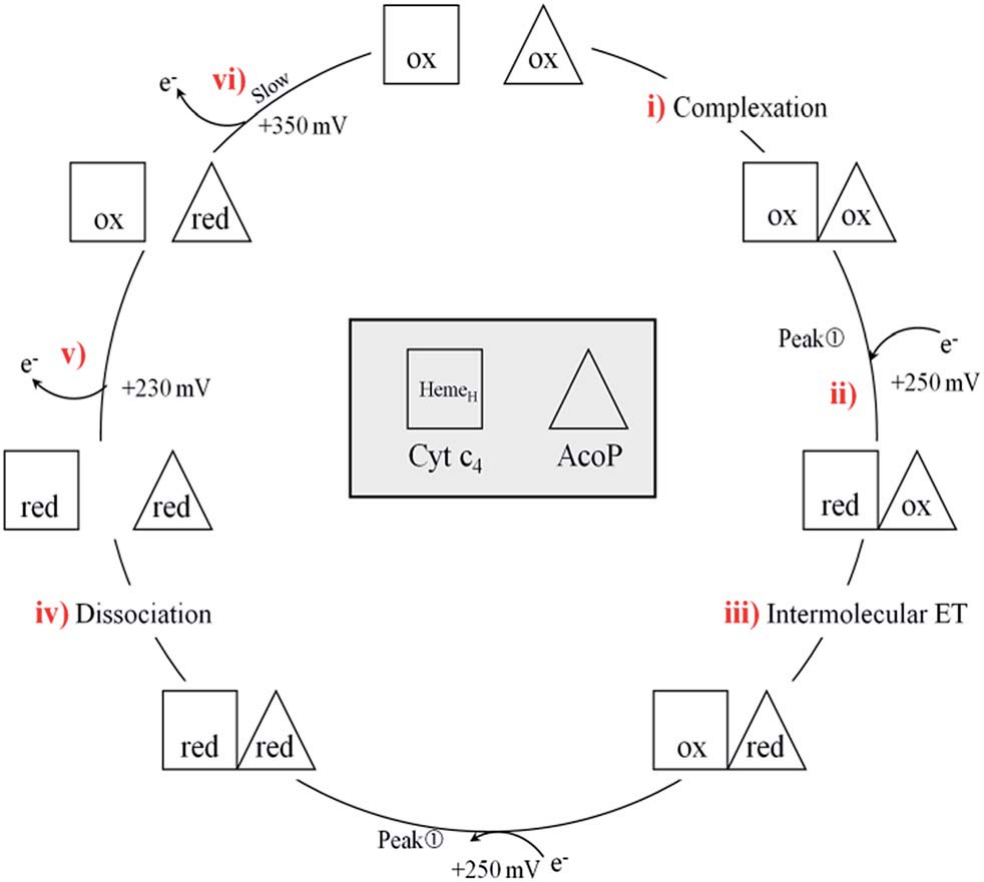
Electrochemical and chemical steps involved in the ET pathway from the oxidized form of the AcoP and Heme_H_ Cyt c_4_ complex to the reduced form explaining the appearance of peak complex to the reduced form explaining the appearance of peak ① observed in observed in Fig. 8.

In a study focusing on the interaction between Cyt *c* and cytochrome *b*_5_ (Cyt *b*_5_), CVs of a mixture of these two redox proteins revealed a pre-peak before the reduction signal for Cyt *b*_5_. It was proposed that this reductive process was due to fast intermolecular ET between reduced Cyt *b*_5_ and oxidized Cyt *c*. Actually, the electrochemical interface was chemically modified so that Cyt *c* could be reduced at the interface only through the electrogenerated reduced form of Cyt *b*_5_.^32^ The situation is somewhat different in the case of AcoP and Cyt c_4_ because the interfacial ET for AcoP is hindered but not completely forbidden. Effectively, oxidation current is noticeable in Fig. 8A in the potential range for AcoP oxidation, which can be attributed to slow interfacial ET. Nevertheless, the typical CV signal we have observed with the protein mixture can also be attributed to intermolecular ET through the complex formed between Cyt c_4_ and AcoP, whose formation is not driven by electrostatic interactions. Indeed, the addition of 400 mM NaCl to the electrolyte did not drastically modify the CV (Fig. 8E). Peak ). Peak ① is thus the result of intermolecular ET between electrogenerated reduced Cyt c is thus the result of intermolecular ET between electrogenerated reduced Cyt c_4_ and oxidized AcoP according to the processes illustrated in Scheme 2. Since we demonstrated that Heme_L_ does not participate in the interaction with AcoP, its independent electrochemical processes are excluded from the ET model. To explain the full CV, we propose the following kinetic steps: (i) complex formation between the oxidized forms of the protein, (ii) heterogeneous reduction of Cyt c_4_ Heme_H_ through favoured interaction of the complex with the PG electrode *via* the versatile adsorbed Cyt c_4_, (iii) reduction of AcoP through intermolecular ET from the reduced Heme_H_ of Cyt c_4_ along peak along peak ①, (iv) dissociation of the complex after the reduction of the proteins, (v) heterogeneous oxidation of Cyt c, (iv) dissociation of the complex after the reduction of the proteins, (v) heterogeneous oxidation of Cyt c_4_, (vi) slow oxidation of AcoP at potentials above +350 mV, and (vii) reassociation of the two oxidized proteins through the complex.

**Fig. 9:**
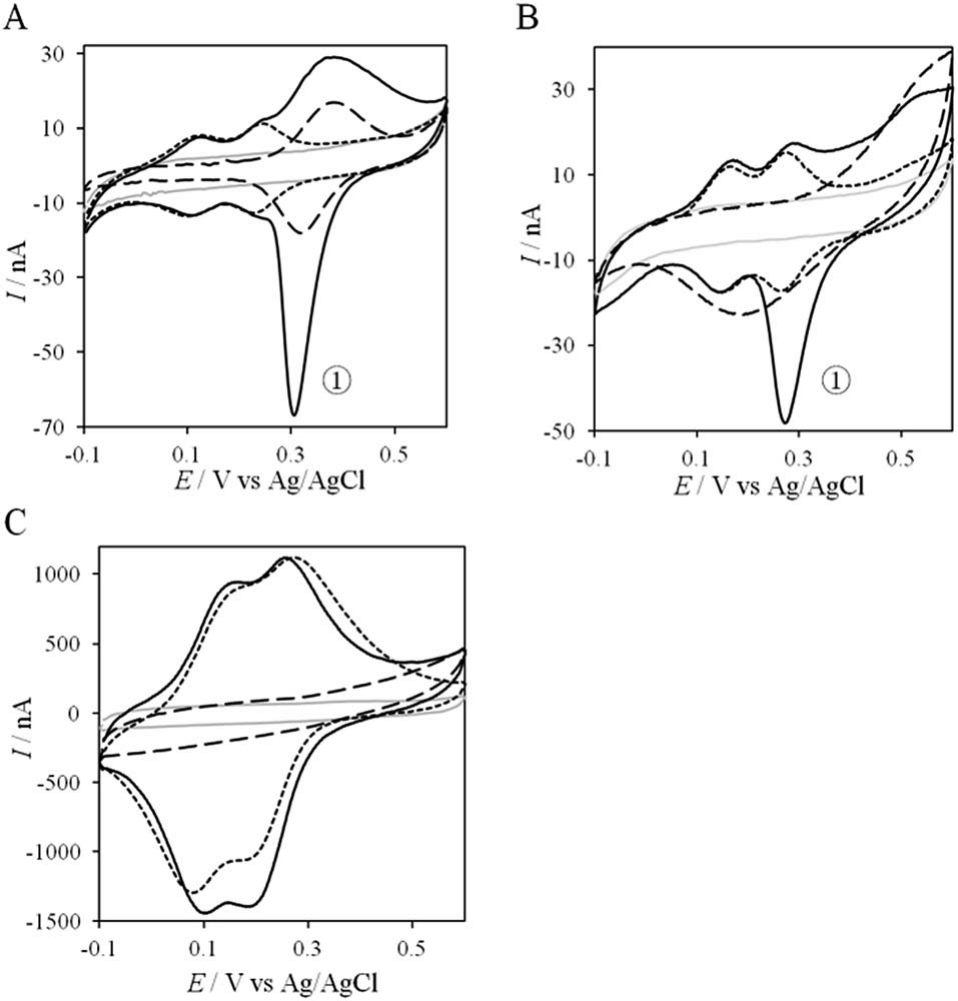
Effect of the scan rate and pH on the electrochemical behavior of the AcoP : Cyt c_4_ complex at a PG electrode in the membrane configuration. CV at 2 mV s^–1^ of AcoP (long dashed line), Cyt c_4_ (short dashed line), and the 1 : 1 mixture of AcoP and Cyt c_4_ (solid line) at (A) pH 4.8 and (B) pH 2.8; (C) CVs as in (A) but at 200 mV s^–1^ and pH 4.8; grey solid lines represent the CVs with no protein in 20 mM NH_4_AC buffer.

If the peculiar electrochemical behavior of the mixture of Cyt c_4_ and AcoP is linked to the formation of a complex that facilitates AcoP reduction, the sweep rate should affect the overall signal. To gain more insight into the redox processes involving the AcoP : Cyt c_4_ mixture, the CVs at 20 mV s^–1^ were compared to CVs obtained at 2 and 200 mV s^–1^. Typical CVs are shown in Fig. 9, where for each sweep rate, the signatures for AcoP and Cyt c_4_ alone and for the mixture are overlaid. At 2 mV s^–1^ (Fig. 9A), the CV of the complex is characterized by two redox waves assigned to Cyt c_4_, with a decrease in the peak height proportional to the sweep rate, as expected for thin layer electrochemistry. As the interfacial ET for AcoP alone is a slow process, the oxidative signal for AcoP at a low scan rate is now visible at the expected potential value. Nevertheless, peak , with a decrease in the peak height proportional to the sweep rate, as expected for thin layer electrochemistry. As the interfacial ET for AcoP alone is a slow process, the oxidative signal for AcoP at a low scan rate is now visible at the expected potential value. Nevertheless, peak ① is still observed, meaning that intermolecular ET is faster than the heterogeneous reduction of AcoP. The experiments described before were carried out at pH 4.8. In the context of an acidophilic chain, it should be more relevant to study the complex electrochemical behavior at a physiological pH. Thus, we recorded the CVs at pH 2.8, in the presence of the AcoP : Cyt c is still observed, meaning that intermolecular ET is faster than the heterogeneous reduction of AcoP. The experiments described before were carried out at pH 4.8. In the context of an acidophilic chain, it should be more relevant to study the complex electrochemical behavior at a physiological pH. Thus, we recorded the CVs at pH 2.8, in the presence of the AcoP : Cyt c_4_ mixture at the PG membrane electrode. Peak mixture at the PG membrane electrode. Peak ① linked to the reduction of AcoP linked to the reduction of AcoP *via* intermolecular ET through Cyt c_4_ was clearly observed (Fig. 9B). As the interfacial ET for AcoP alone is very slow at this low pH, AcoP reduction can only proceed through intermolecular ET *via* Cyt c_4_. This is an additional proof of the occurrence of an efficient ET complex between the two partners.

**Fig. 10:**
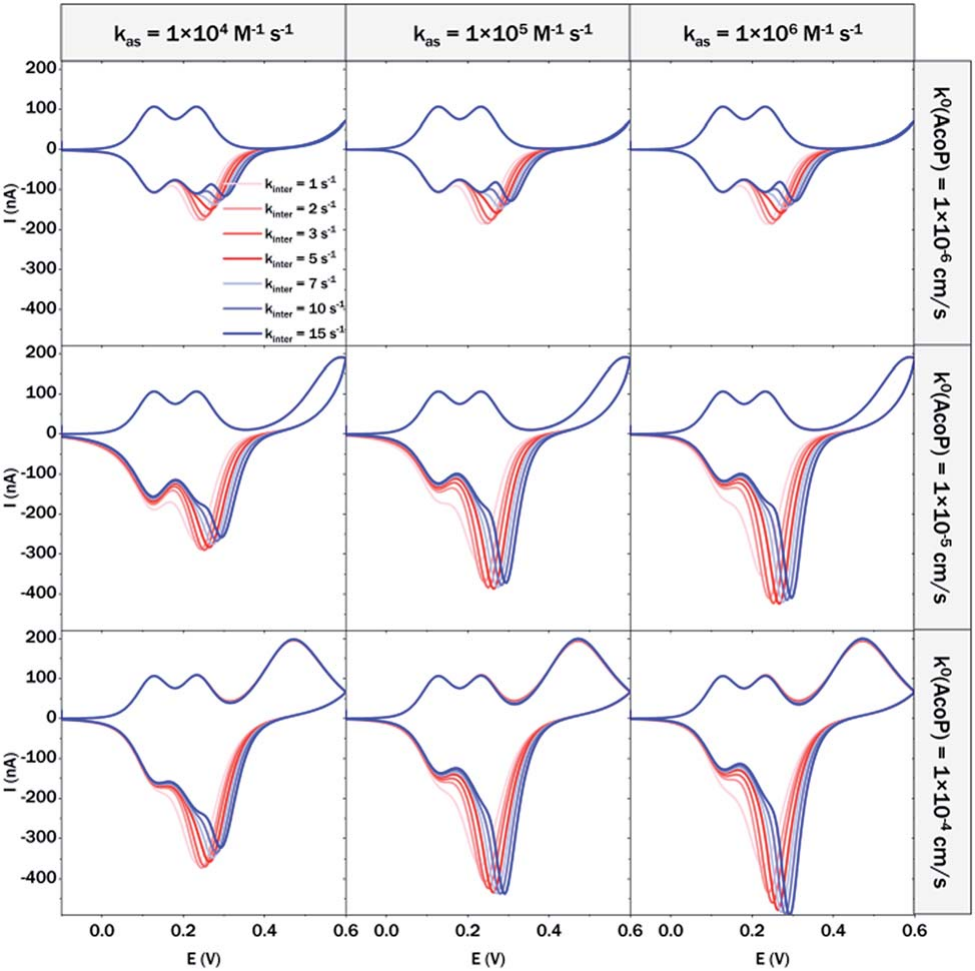
Modelling of the CVs showing the influence of three different constants, the heterogeneous electron transfer constant of AcoP (*k*^0^), the kinetic rate constant of the complex formation (*k*_as_) and the constant of the intermolecular electron transfer within the complex (*k*_inter_), on the shape of the simulated voltammograms at 20 mV s^–1^ (capacitive current is excluded).

At 200 mV s^–1^, only the redox waves for Cyt c_4_ are observed (Fig. 9C). At this high speed, one or several kinetic processes may be limiting steps precluding the peak ). At this high speed, one or several kinetic processes may be limiting steps precluding the peak ① observation: slow AcoP oxidation, slow complex formation or slow intermolecular electron transfer within the complex. To verify the influence of each process and confirm complex formation, numerical modeling of the CVs as described in the ESI (Fig. S8 observation: slow AcoP oxidation, slow complex formation or slow intermolecular electron transfer within the complex. To verify the influence of each process and confirm complex formation, numerical modeling of the CVs as described in the ESI (Fig. S8†) was performed at 2, 20 and 200 mV s^–1^ as a function of the heterogeneous electron transfer constant of AcoP (*k*^0^), the kinetic rate constant of the complex formation (*k*_as_) and the constant of the intermolecular electron transfer within the complex (*k*_inter_). Fig. 10 illustrates one such simulation at 20 mV s^–1^.

The shapes of the theoretical CV curves tend to match those of the experimental ones, confirming the intermolecular ET through a complex between adsorbed Cyt c_4_ and AcoP. The constant of the intermolecular electron transfer has the strongest influence on the potential of peak and AcoP. The constant of the intermolecular electron transfer has the strongest influence on the potential of peak ① which only slightly varies as a function of the other two constants. This can be used for the estimation of this constant. Based on the simulation at the three different scan rates, and hypothesizing a fast complex formation which is thermodynamically favoured, the intermolecular electron transfer within the complex could be estimated to occur with a rate of 3–5 s which only slightly varies as a function of the other two constants. This can be used for the estimation of this constant. Based on the simulation at the three different scan rates, and hypothesizing a fast complex formation which is thermodynamically favoured, the intermolecular electron transfer within the complex could be estimated to occur with a rate of 3–5 s^–1^. This value is smaller than other values estimated from photoinduced intracomplex ET between Cyt *f* and Cyt c_6_ from *Chlamydomonas reinhardtii* (9 × 10^2^ s^–1^)^43^ or intracomplex ET obtained by laser excitation of ruthenium-labeled Cyt *b*_5_ and cyt *c* (4 × 10^5^ s^–1^).^44^ Our intermolecular ET between AcoP and Cyt c_4_ is in the same range as the value reported for ET between *Pseudomonas aeruginosa* cyt c_551_ and *Silene pratensis* plastocyanin obtained by stopped-flow measurements (5.7 s^–1^).^45^ Two hypotheses may explain the slow intermolecular ET rate between AcoP and Cyt c_4_: (i) the adsorption of Cyt c_4_ at the electrode surface impairs the optimal ET complex; (ii) AcoP has additional functions other than accepting electrons from Cyt c_4_.

## Experimental

### Chemicals and biomaterials

(+)-Sodium l-ascorbate (ascorbate), sodium dithionite, potassium hexachloroiridate(iv) (K_2_IrCl_6_), CM sepharose fast flow resin (CMC resin), a HiLoad 26/10 SP Sepharose HP column (SP Sepharose column), a Mono S™ 5/50 GL column (MonoS column), ethylenediaminetetraacetic acid disodium salt dehydrate (EDTA), DNAse I from bovine pancreas, anti-protease (fast protease inhibiter tablets), *n*-dodecyl β-*d*-maltoside (DDM), sodium acetate (NaAC), ammonium acetate (NH_4_AC), 1-butanethiol (99%) (BT), 4-aminothiophenol (97%) (4-ATP), 6-mercaptohexanoic acid (90%) (MHA), 30% H_2_O_2_, NaOH, 98% H_2_SO_4_, absolute ethanol (EtOH), acetic acid (96%), and sodium chloride were bought from Sigma-Aldrich and used as received. All aqueous solutions were prepared with Milli Q (18.2 MΩ cm) water.

Recombinant AcoP without its transmembrane segment was expressed and purified from *Escherichia coli* as described previously.^14^ AcoP protein concentration was determined by UV-vis absorbance spectroscopy, measuring the absorption intensity at 280 nm and using a sequence-derived theoretical molar extinction coefficient of 25 440 M^–1^ cm^–1^. Purified AcoP was stored in 50 mM NaAC buffer at pH 4.8, containing 0.005% (w/v) DDM. When mentioned, AcoP was dialysed against 10 or 20 mM NH_4_AC buffers adjusted to different pH values, pH 2.8, pH 3.5, pH 4.8 and pH 7.

Cyt c_4_ was expressed as described previously in 100 l *E. coli* cultures^9^,^34^ using three separation steps on carboxymethyl cellulose, SP-Sepharose and monoS columns. The enriched fractions containing Cyt c_4_ in 80 mM NH_4_AC buffer at pH 4.8 were pooled and dialysed against 80 mM NH_4_AC buffer at pH 2.8, followed by another dialysis at pH 4.8. This pH variation allowed further elimination of *E. coli* acid unstable proteins. When mentioned, purified Cyt c_4_ was dialysed against 10 or 20 mM NH_4_AC buffers adjusted to different pH values, pH 2.8, pH 3.5, pH 4.8 and pH 7.

Cytochrome *c* (Cyt *c*) from an equine heart (≥95%, SDS-PAGE) was bought from Sigma-Aldrich. Cyt *c* was diluted in NH_4_AC 20 mM buffer at pH 4.8.

### Protein modelling

For cupredoxins, being well known, several structures have been solved which serve as templates for modelling the globular soluble domain of AcoP. Here we used the Phyre2, Swiss-model and I-TASSER servers^47^ obtaining similar results. We note that, given the high homology with the templates, this model is expected to be of reasonable quality along most of the sequence.^48^,^49^ For Scheme 1, AcoP's TM helix was built manually by threading its sequence as an ideal helix. The model of Cyc2 in Scheme 1 is based on I-TASSER homology modelling guided by coevolution-based restraints as described in ref. 50. For Cyt c_4_ of *Acidithiobacillus acidophilus* (pdb: ; 1H1O) and Cyt c_4_ of *Pseudomonas stutzeri*, we used available X-ray structures (pdb: ; 1H1O and ; 1ETP, respectively).

To study protein electrostatics, AcoP models and Cyt c_4_ structures were prepared with the PDB2PQR-Propka servers, using the Amber force field to assign electrostatic charges at the surface of the proteins and the Parse force fields to determine the dipole moments of proteins with PDB2PQR-Propka servers.^51^ Dipole moments were also determined from the pqr output files using the Protein Dipole Moments Server.^52^ Electrostatic charges at the surface of proteins and surface hydrophobicity were illustrated using PyMOL (The PyMOL Molecular Graphics System, Version 1.8 Schrodinger, LLC).

### Polyacrylamide gels

#### SDS-PAGE analysis

Samples were mixed with 4× Laemmli buffer (Laemmli at 1× contains 62.5 mM Tris (pH 6.8), 12.5% glycerol, 2% SDS, 5% (v/v) β-mercaptoethanol, and 0.01% bromophenol blue) and incubated at 100 °C for 10 min. The samples were then run in 15% SDS-polyacrylamide gel. Proteins were visualized by Coomassie Blue staining or PageBlue™ protein staining (Thermo Fisher).

#### Modified-native-PAGE analysis

Samples were mixed with DDM at a final concentration of 0.01% and incubated at room temperature for 45 min. The treated samples were mixed with 2× modified Laemmli buffer (1× contains 125 mM Tris (pH 6.8), 25% glycerol, 0.0175% SDS, and 0.01% bromophenol blue) and charged directly on 15% Tris–glycine native gel containing 0.0175% SDS. The gels were run in Tris–glycine running buffer with 0.0175% SDS. Proteins were visualized by Coomassie Blue or PageBlue staining.

#### N-Terminal sequence determination

N-Terminal sequence determination was performed by stepwise Edman degradation using an automatic sequencer model PPSQ 31B (Shimadzu, Kyoto, Japan).

### Electrochemical measurements

Cyclic voltammetry (CV) and square wave voltammetry (SWV) were performed using an Autolab PGSTAT30 potentiostat analyzer controlled by Nova software (Eco Chemie). The electrochemical cell was equipped with three electrodes, a pyrolytic graphite (PG) or a gold electrode as the working electrode (*S* = 0.07 cm^2^), a platinum wire as an auxiliary electrode and an Ag/AgCl electrode as the reference electrode. All potentials are quoted *vs.* the Ag/AgCl reference electrode here. Potentials *versus* NHE can be obtained by adding 210 mV to the reported potentials. 20 mM NH_4_AC buffer was used as the electrolyte. All the electrochemical measurements were made at least in triplicate at 25 °C.

The PG electrode surface was renewed by polishing with fine sand paper (P1200), and then briefly sonicated to remove free carbon particles. The membrane electrode configuration was used to entrap 2 μl of protein sample in a thin layer between the electrode and a dialysis membrane of suitable cutoff.^53^ The gold surfaces were cleaned with “piranha” solution (3H_2_SO_4_ 98% : 1H_2_O_2_ 30%) for 4 min and rinsed extensively with water and later with ethanol. Self-assembled monolayers (SAMs) were formed by immersing the gold surfaces in 5 mM ethanol solutions of 4-ATP or MHA or BT for 18 hours. The surfaces were then cleaned with ethanol to remove all organic contaminants.

### Spectroscopies

Circular Dichroism (CD) spectra of 15 μM proteins were recorded on a Jasco J-715 spectropolarimeter at 298 K in a 1 mm path length cell. Spectra were averaged from five scans and normalized for any variation in protein concentrations measured at 280 nm by optical spectroscopy using a Cary 50 Bio (Varian).

UV-vis spectra were recorded on a Cary 50 Bio (Varian) spectrophotometer. Protein samples (AcoP or Cyt c_4_) were used at 15 μM, for a scan recorded from 200 nm to 1000 nm to follow metal center properties. When mentioned, oxidation or reduction of the samples was performed using an equimolar amount of protein and potassium hexachloroiridate(iv) (K_2_IrCl_6_) or ascorbate. Ascorbate can be removed for further analysis by using a desalting column (PD-10 column, GE Healthcare). Because iridium is a strong oxidizing agent that irreversibly modifies key residues, we used “as prep” AcoP which corresponds to ∼40% of the oxidized form.^14^

X-band electron paramagnetic resonance (EPR) spectra were recorded using a Bruker-Biospin EleXsys E500 spectrometer equipped with a standard rectangular Bruker EPR cavity (ER4102ST) connected to an Oxford Instruments helium flow cryostat (ESR900).

For polarization modulation-infrared reflection-adsorption spectroscopy (PM-IRRAS), the modified dried gold surface was placed at room temperature in the external beam of the FT-IR instrument on a Nicolet Nexus 870 FT-IR spectrometer (Madison, WI). The experimental set-up has been reported in a previous paper.^54^ The PMIRRAS spectra were recorded at 8 cm^–1^ resolution, with the co-addition of six hundred scans.

ATR-FTIR spectra were recorded on a Nicolet 6700 FT-IR spectrometer (Nicolet Instrument, Madison, WI) equipped with a liquid nitrogen cooled mercury–cadmium–telluride detector (Thermo Fisher Scientific, San Jose, CA), with a spectral resolution of 4 cm^–1^. Two hundred interferograms were co-added.

## Conclusions

**Scheme 3:**
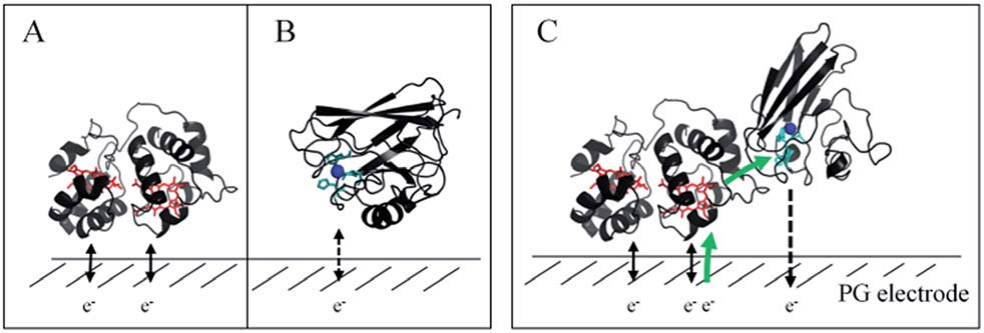
Proposed model of ET between redox proteins and the electrode surface observed on PG electrodes (see Fig. 8). Dashed lines represent PG electrodes. Structures of Cyt c_4_ or predicted AcoP are presented in grey ribbons with their respective redox centers, hemes (in red) and copper (in blue). (A) Interfacial ET between the electrode and the two heme centers, a reversible process, illustrated by black arrows; (B) slow interfacial ET between the AcoP copper center and the electrode shown by a dashed arrow; (C) additional intermolecular ET pathway within the AcoP : Cyt c_4_ complex depicted by a green arrow. This complex has been modelled with the HADDOCK web server^46^ by driving the docking with the constraint based on AcoP interacting with Heme_H_ of Cyt c_4_.

In this study, we demonstrated the formation of a complex between AcoP and Cyt c_4_, two proteins of an acidophilic bacterial respiratory chain. We used an electrode interface to mimic AcoP and Cyt c_4_ as putative redox partners in the energy chain. We found that electrostatic interactions do not drive efficient ET from the AcoP copper centre to the electrodes. In contrast, we showed that Cyt c_4_ is highly versatile, displaying ET at the two heme redox centres on varied types of surfaces, either positive, negative or hydrophobic. This suggests that Cyt c_4_ could be a hub that controls the rate of electrons towards different routes, depending on the metabolic or environmental states. We also demonstrated an intermolecular ET between AcoP and Cyt c_4_, allowing the reduction of AcoP through the high potential heme of Cyt c_4_ (Scheme 3). Thanks to modeling of the electrochemical signals, we were able to quantify the intermolecular ET rate within the complex. Although the results for the mixture suggest a likely directionality of ET from Cyt c_4_ to AcoP, the value of the intermolecular rate constant opens a new direction toward the physiological role of AcoP.

The electrochemical setup developed in this work allowed us to reconstitute on electrodes a portion of a respiratory chain and to quantify ET rates using small amounts of proteins and with the possibility to very easily tune the protein environment. We propose that this method could serve to get thermodynamic and kinetic data on other redox proteins, as well as between two interacting redox partners. Furthermore, for membrane proteins, the use of electrochemical interfaces appears more appropriate to mimic their physiological environment, in comparison with classical solution-state stopped-flow analysis.

Using the same strategy and by integrating the other partners of the respiratory chain, we should be able in the future to complete the understanding of the energy chain of *A. ferrooxidans.* For upstream Cyt c_4_, it was demonstrated that Rus was interacting with Cyt c_4_*via* Heme_H_.^7^ Rus and AcoP are both cupredoxins of high redox potential. The purpose served by AcoP is not clear as Rus could be sufficient to shuttle electrons in the periplasm. Their stoichiometry (Rus is highly abundant)^55^ and localization (membrane anchored for AcoP *versus* periplasm for Rus) might drive their specific function and explain their co-occurrence. It should be interesting to determine whether competition exists between the two cupredoxins in the respiratory chain, and under which conditions*.* Downstream, the question of the electron transfer to the terminal oxidase C*c*O should be addressed. In most known biological systems, Cyt *c* has been described as the electron donor to the C*c*O.^56^ However, in some biological systems, cupredoxins have been shown to replace Cyt *c* as electron donors to terminal enzymes depending on the metal occurrence in the bacterial environment.^57^ In the case of *A. ferrooxidans*, AcoP and Cyt c_4_ being under the control of the same promoter, and hence expressed under the same conditions, might both be able to transfer electrons to the CcO. We can hypothesize that AcoP may act as a second entry for electrons, while protecting the CcO copper center exposed to the acidic environment.^11^ Further studies are required to answer such questions.

For a long time *A. ferrooxidans* has attracted the interest of industries regarding its use in bioleaching processes. More recently, its advantage as a biocathodic catalyst in microbial fuel cells^3^ expanded the importance of its study, which emphasizes the need to better describe how this bacterium finds energy to grow using iron, a low energetic substrate. Even more interestingly, proteins in this ET chain share the common features of having a high redox potential. Such properties can be of great importance for ecologically friendly devices such as enzymatic fuel cells. These fuel cells are cathode-limited by the low oxygen affinity of currently used multicopper oxidases. CcO could be an alternative, but the most widely characterized CcOs display low redox potentials, a limitation that *A. ferrooxidans* C*c*O could overcome.

## Conflicts of interest

There are no conflicts to declare.

## Supplementary Material

Supplementary information
