# Supplementary material for: Electron transfer in an acidophilic bacterium: interaction between a diheme cytochrome and a cupredoxin
Source: Chem Sci. 2018 May 1;9(21):4879–91. doi: 10.1039/c8sc01615a (PMC5982212; doi:10.1039/c8sc01615a)
Supplement: Supplementary file 1 [file SC-009-C8SC01615A-s001.pdf]

Electronic Supplementary Material (ESI) for Chemical Science.
This journal is © The Royal Society of Chemistry 2018

Electronic supplementary information

Electron transfer in an acidophilic bacterium: interaction between a diheme cytochrome and a cupredoxin

X. Wang, [a] M. Roger, [b] R. Clément, [a] S. Lecomte, [c] F. Biaso, [a] L. A. Abriata, [d] P. Mansuelle, [e] I. Mazurenko, [f] M.T. Giudici-Orticoni, [a] E. Lojou, *[a] M. Ilbert*[a]

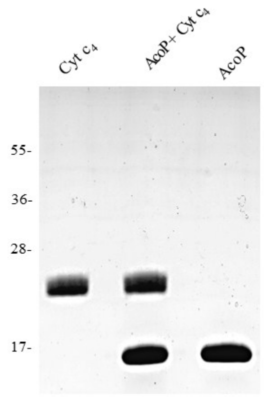

Fig. S1 SDS-PAGE gel where 5 µL of 100 µM of Cyt c_4, AcoP and AcoP:Cyt c_4 complex were loaded. Numbers in kDa on the gels correspond to the molecular weight of the ladder.

A

Proteins	Edman Sequencing cycles				
	Cycle 1	Cycle 2	Cycle 3	Cycle 4	Cycle 5
Cyt c_4 theoretical	A	V	G	S	A
**Cyt c_4 experimental**	**A**	**V**	**G**	**S**	**A**
AcoP theoretical	L	P	N	P	S
**AcoP experimental**	**L**	**P**	**N**	**P**	**S**
AcoP + Cyt c_4 experimental	**LA**	**PV**	**NG**	**PS**	**SA**

B

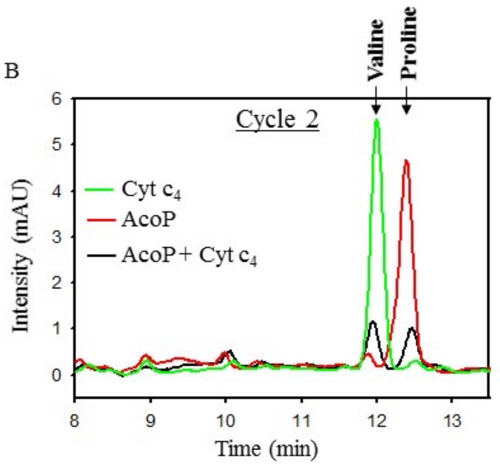

Fig. S2 N terminal sequencing by Edman degradation. A) Amino acid residues identified by Edman degradation on protein bands extracted from a Western Blot membrane. In green is presented the result obtained from a band when only Cyt c_4 was loaded on gel, in red is presented the result obtained when only AcoP was loaded on a gel. When the complex Cyt c_4:AcoP was loaded on a gel, an additional band (labeled * in Fig. 1) appears, after Western blot this band was analyzed by Edman degradation, results obtained are presented in the table (in black). For each cycle, quantities of each residue were of the same order making impossible to assign a residue to a peculiar sequence and thus supporting the hypothesis of an equimolar ratio between the two proteins. B) One representative cycle (Zoom of cycle 2) is shown. In the band (*) where AcoP and Cyt c_4 has been loaded together on a gel (black curve), the same amount of valine and proline are detected demonstrating the presence of both proteins Cyt c_4 and AcoP in this band in comparison with AcoP alone (in red, Proline) and Cyt c_4 alone (in green, Valine).

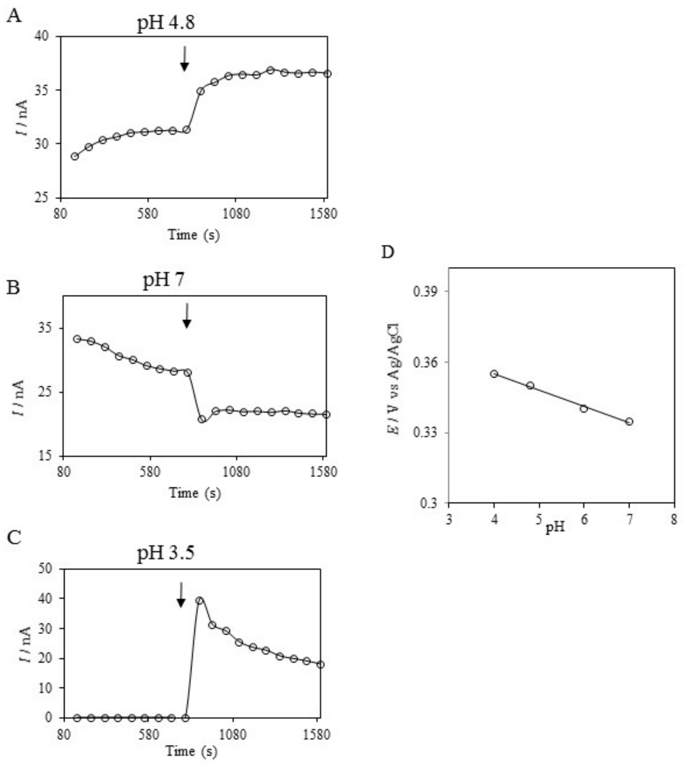

Fig. S3 (A-C) Stability of AcoP anodic peak current measured with continuous CV at pH 4.8 (A), pH 7 (B) and pH 3.5 (C) before and after 200 mM NaCl addition denoted by the arrow. (D) Relationship between E_m and pH. The redox potential of AcoP varies with a slope of 7 mV/pH, showing that the ET process is not coupled to a proton transfer, but instead protonation of some amino acid residues in the vicinity of the Cu center is involved.

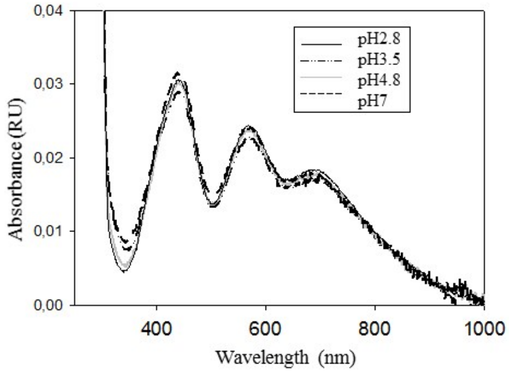

Fig. S4 UV-Vis spectra of AcoP (60 μM) at various pHs.

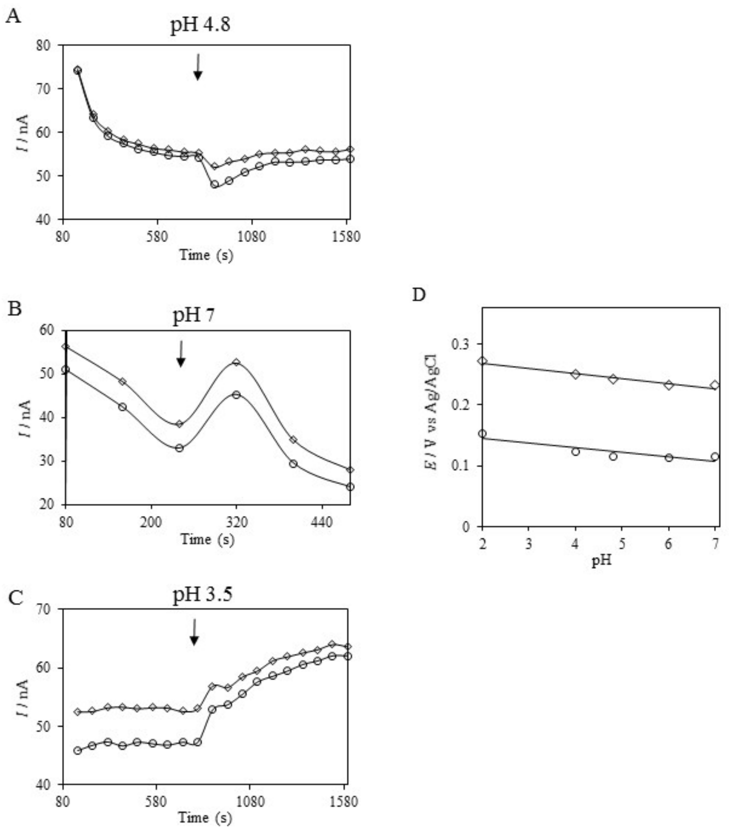

Fig. S5 (A-C) Stability of $Heme_L$ (circles) and $Heme_H$ (diamond) anodic peak currents of Cyt c_4 measured with continuous CV at pH 4.8 (A), pH 7 (B) and pH 3.5 (C) before and after 200 mM NaCl addition denoted by the arrow. (D) Relationship between of E_m and pH.

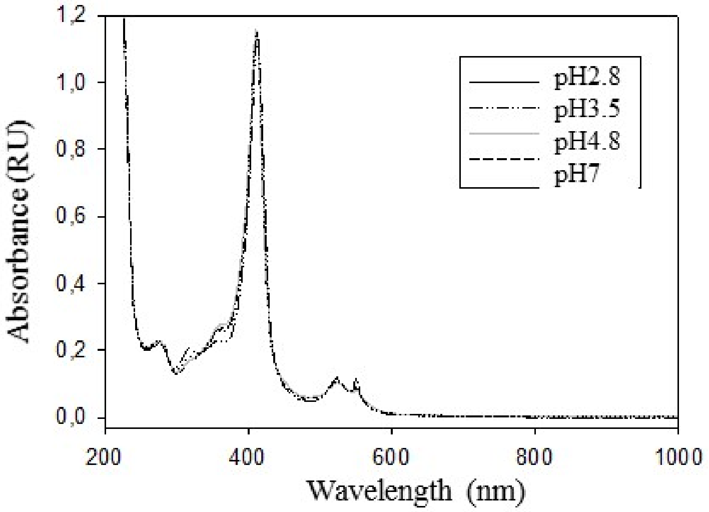

Fig. S6 UV-Vis spectra of Cyt c_4 (60 μM) at various pHs in 20 mM NH_4AC buffer.

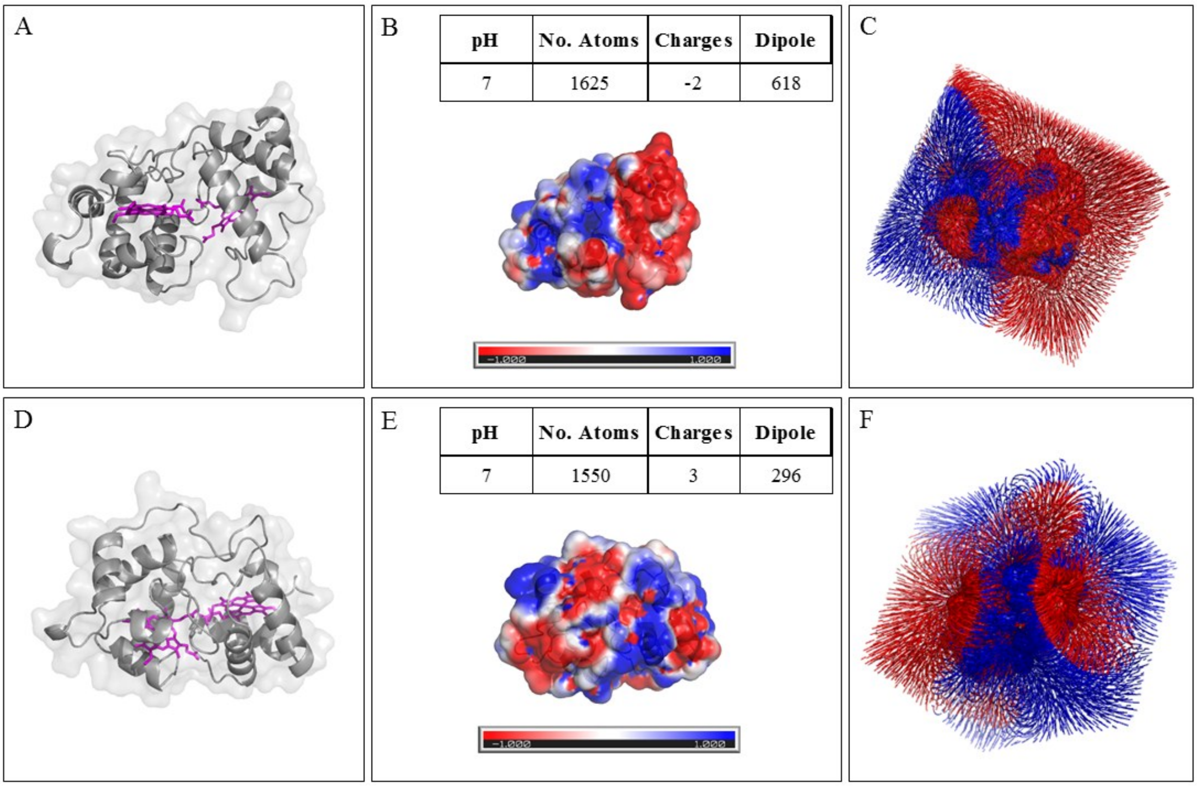

Fig. S7 Electrostatic surface charges of Cyt c_4 from *A. ferrooxidans* compared to Cyt c_4 from *P. stutzeri*. Structures of Cyt c_4 of (A) *P. stutzeri* (PDB: 1ETP) [1], and (B) *A. ferrooxidans* (PDB: 1H1O) [2]. The two hemes are colored in magenta. Comparison of the values at pH 7 of the dipole moment and surface charges of Cyt c_4 from *P. stutzeri* (B, C) and from *A. ferrooxidans*. (E, F).

2 mV/s

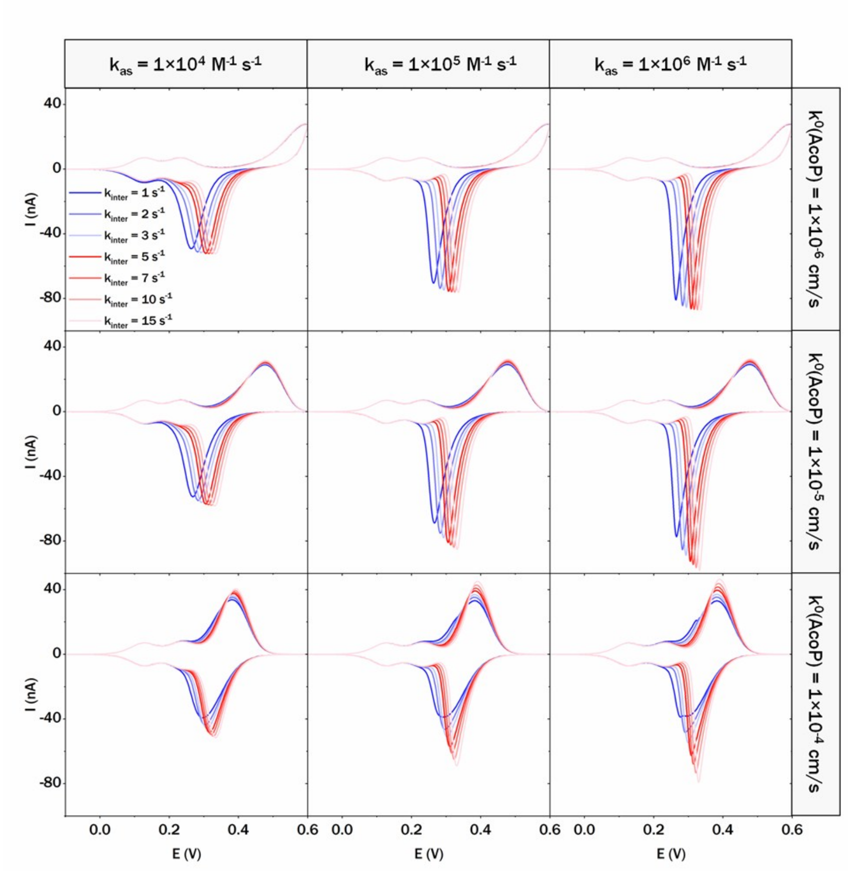

200 mV/s

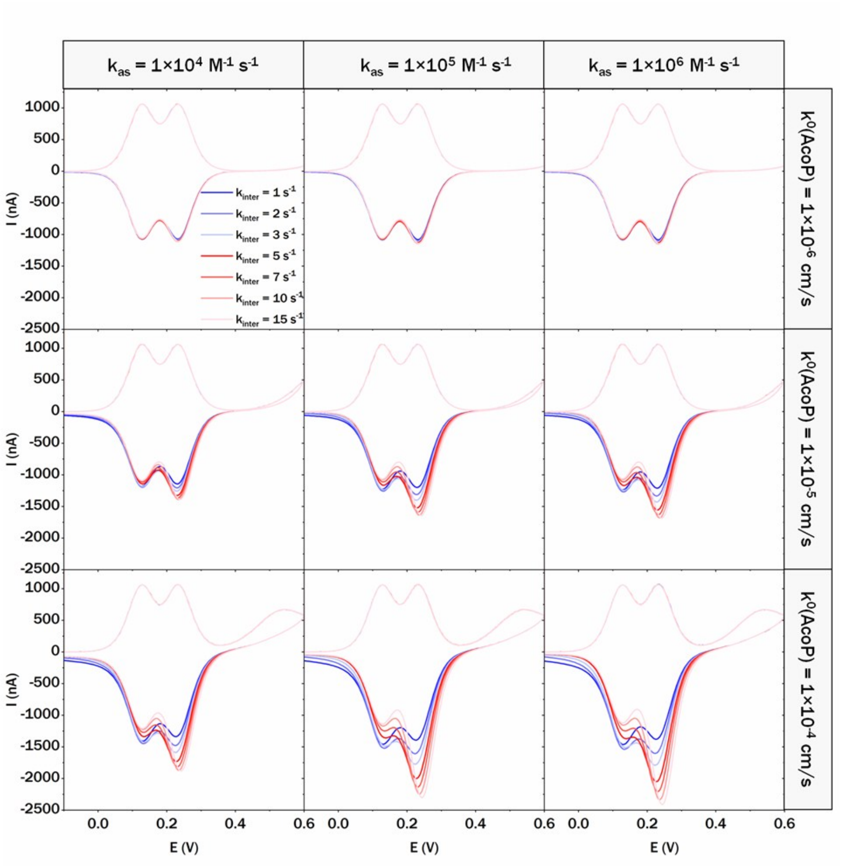

Fig. S8 Modelling of the CVs showing the influence of three different constants, heterogeneous electron transfer constant of AcoP (k^0), kinetic rate constant of the complex formation (k_{as}) and the constant of the intermolecular electron transfer within the complex (k_{inter}) on the shape of simulated voltamograms at 2 and 200 mV.s^{-1} (excluding the capacitive current).

For the modelling, the following reactions are assumed to take place ("s" superscript depicts surface adsorbed species):

1	Cyt_r^s - ē → Cyt_o^s (Heme L)	Electrochemical process at the surface	E_1 = 0.125 V	k^0_{Cyt} (s^{-1})
2	Cyt_r^s - ē → Cyt_o^s (Heme H)	Electrochemical process at the surface	E_2 = 0.235 V	k^0_{Cyt} (s^{-1})
3	$AcoP_r$ - ē → $AcoP_o$	Electrochemical process in the thin layer at the electrode	E_3 = 0.35 V	k^0_{AcoP} (cm s^{-1})
4	Cyt_o^s + $AcoP_o$ → $C_oA_o^s$	Complex formation at the electrode surface		k_{as} (L mol^{-1} s^{-1})
5	$C_oA_o^s$ + ē → $C_rA_o^s$		E = E_2	k^0_{Cyt} (s^{-1})
6	$C_rA_o^s$ → $C_oA_r^s$	Intermolecular electron transfer process		k_{inter} (s^{-1})
7	$C_oA_r^s$ + ē → $C_rA_r^s$		E = E_2	k^0_{Cyt} (s^{-1})
8	$C_rA_r^s$ → Cyt_r^s +$AcoP_r$	Complex dissociation		k_{dis} (s^{-1})

The reactions 1-3, 5, 7 are assumed to proceed according to the Butler-Volmer kinetics giving raise to the current:

$$I = nFk^0 A\left([Red]\exp\left(\frac{(n-\alpha)F}{RT}(E-E^0)\right) - [Ox]\exp\left(\frac{(n-\alpha)F}{RT}\cdot(E-E^0)\right)\right)$$

where [Red] corresponds to surface concentration of the reduced forms ($AcoP_{red}$ or Cyt_r^s alone or within the complex), [Ox] corresponds to surface concentration of the oxidized forms ($AcoP_o$ or Cyt_o^s alone or within the complex), k^0 are the corresponding rate constants (expressed in cm s^{-1} for diffusing species, or in s^{-1} for adsorbed species), E^0 are the formal potentials for the corresponding reactions.

The reactions 4, 6, 8 are considered as quasi-irreversible, i.e. only forward rate constants are taken into account. This is particularly true for the reaction 6 since the redox potential difference between the Cu-centre of AcoP (0.35 V) and the $Heme_H$ of Cyt c_4 (0.235 V) is too large to enable the backward electron transfer. The rate of the reaction 8 should not influence the voltammograms, and it is supposed to be fast enough since the second cycle is almost identical to the first one. Then the following rate equations can be expressed:

$$v_4 = k_{as}\left[Cyt_o^s\right]\left[AcoP_o\right]$$

$$v_6 = k_{inter}\left[C_r A_o^s\right]$$

$$v_8 = k_{dis}\left[C_r A_r^s\right]$$

This set of electrochemical and rate equations was introduced and solved in Comsol Multiphysics® 5.3a using 1D-model corresponding to the thickness of diffusion layer under the membrane (30 µm). The following parameters were fixed in the model:

Surface area of the electrode	A	0.071 cm^2
Surface rate constant of Cyt c_4	k^0_{Cyt}	10^6 s^{-1}
Complex dissociation constant	k_{dis}	10^3 s^{-1}
AcoP diffusion coefficient	D	10^6 cm^2/s
Apparent Cyt c_4 surface coverage	Γ	50 pmol/cm^2

The model was solved for different combinations of k^0_{AcoP}, k_{as} and k_{inter} at 2, 20 and 200 mVs^{-1}.

It should be noted that, despite close similarity, we were not able to reproduce exactly the shape of the experimental voltamograms due to a number of reasons: a) the exact thickness of the diffusion layer under the membrane is unknown, and may vary from one experiment to another; b) although Cyt c_4 is considered to be adsorbed, some quantity may be left in the thin layer solution and may contribute to a non-negligible diffusion current; c) the presence of adsorbed Cyt c_4 may influence the electrochemical properties of AcoP.

Nevertheless, the general trend is clearly visible from these sketches:

- The constant of the complex formation has rather weak influence on the shape of voltamograms, provided that it is high enough, notably compared to the heterogeneous electron transfer constant of AcoP
- The heterogeneous electron transfer constant of AcoP determines the amount of complex formed, i.e. the height of the peak ①, since only the oxidized form of AcoP participates in the complex formation.
- The rate of the intermolecular electron transfer has the strongest influence on the potential of the peak ① which only slightly varies as a function of the two other constants.

References

[1] Kadziola, A., Larsen, S. Crystal structure of the dihaem cytochrome c4 from *Pseudomonas stutzeri* determined at 2.2 Å resolution. *Structure* **5**, 203-216 (1997)

[2] Abergel, C., Nitschke, W., Malarte, G., Bruschi, M., Claverie, J.-M., Guidici-Orticoni, M.-T. The Structure of *Acidithiobacillus ferrooxidans* C(4)-Cytochrome. A Model for Complex-Induced Electron Transfer Tuning. *Structure* **11**, 547(2003)
